# Supplementary material for: Intestinal Pgc1α ablation protects from liver steatosis and fibrosis
Source: JHEP Rep. 2023 Jul 19;5(11):100853. doi: 10.1016/j.jhepr.2023.100853 (PMC10597770; doi:10.1016/j.jhepr.2023.100853)
Supplement: Multimedia component 1 [file mmc1.pdf]

Supplementary Material

Intestinal Pgc1α ablation protects from liver steatosis and fibrosis

Elena Piccinin [1,2], Maria Arconzo [2], Maria Laura Matrella [1], Marica Cariello [2], Arnaud Polizzi [3], Yannick Lippi [3], Justine Bertrand-Michel [4], Hervé Guillou [3], Nicolas Loiseau [3], Gaetano Villani [1], Antonio Moschetta[2,5]*

[1] Department of Translational Biomedicine and Neuroscience (DiBraiN), University of Bari "Aldo Moro", Bari, Italy
[2] Department of Interdisciplinary Medicine, University of Bari "Aldo Moro", Bari, Italy
[3] Toxalim (Research Center in Food Toxicology), INRAE, ENVT, INP-PURPAN, UMR 1331, UPS, Université de Toulouse, Toulouse, France
[4] MetaboHUB-MetaToul, National Infrastructure of Metabolomics and Fluxomics, Toulouse, France
[5] INBB, National Institute for Biostructures and Biosystems, Rome, Italy

Table of contents

Supplementary Methods 2
Supplementary Figure 1 8
Supplementary Figure 2 9
Supplementary Figure 3 10
Supplementary Figure 4 11
Supplementary Figure 5 12
Supplementary Table 1 13
Supplementary Table 2 13
Supplementary References 14
CTAT methods **Errore. Il segnalibro non è definito.**

Supplementary Methods

Intestinal Permeability Assay

In vivo intestinal permeability was assessed using fluorescein isothiocyanate (FITC)-conjugated dextran (Sigma-Aldrich, USA; molecular mass 3–5 kDa). Mice were gavaged with 0.6mg/g body weight of FITC. After 4h blood was collected via submandibular puncture, and serum was collected by centrifugation. Serum fluorescence intensely correlating with intestinal permeability was measured in serum with the Microplate fluorometer VICTORTM EnLiteTM (PerkinElmer, Italy). Serum FITC concentration was calculated for each sample.

Glucose tolerance and insulin tolerance tests

For glucose tolerance tests, mice were fasted overnight before receiving an oral gavage with glucose at 2g/kg body weight. Blood glucose levels were measured before glucose administration (t=0) and then at times 15, 30, 60, and 120 minutes. For insulin tolerance tests, mice were fasted for 6h prior intraperitoneally injected with insulin at 0.5U/kg body weight. Blood glucose levels were measured before insulin administration (t=0) and then at times 15, 30, 60, and 120 minutes.

Insulin and GLP-1 assays

Mice were fasted overnight before receiving an oral gavage with glucose at 2g/kg body weight. Blood was collected from the submandibular vein a time 0, 15, and 30 minutes using Microvette serum tubes (Starsted, Germany). Insulin was assessed using Mouse Insulin ELISA (Mercodia, Sweden), following the manufacturer's instructions. GLP-1 levels were measured with Multi Species GLP-1 Total ELISA (Merck, Germany) following the manufacturer's instructions.

Oral lipid tolerance test

Mice were fasted overnight before receiving an oral gavage of intralipid 20 (Sigma-Aldrich, USA) 10mL/kg body weight. Blood was then collected from the tail vein and centrifuged (1000×g, 10 min at 4°C) to obtain plasma before and at 60, 120, 180, and 240 minutes after the administration of the oil. Plasma triglyceride levels were immediately measured using a commercial kit (Sentinel Diagnostic, Italy).

Cholesterol transport assay

Intestinal uptake of cholesterol was measured using TopFluor-cholesterol (Avanti Polar Lipids, Alabaster, AL) in mice fed a chow diet. To mimic intestinal lipid processing, a lipid mixture of TopFluor-cholesterol (8 µg/gm body weight), phospholipids (Sigma-Aldrich, St. Louis, MO), and taurocholate (Sigma-Aldrich, St. Louis, MO) was obtained, generating a so-called a bile model [1]. To this mixture, methyl-β-cyclodextrin (Sigma-Aldrich, St. Louis, MO) was added since it is necessary to deplete already present plasma membrane cholesterol [2]. Blood was collected from the submandibular vein using Microvette serum tubes (Starsted, Germany) at times 0 and 8 hours. Serum fluorescence was read at Microplate fluorometer VICTORTM EnLiteTM (PerkinElmer, Italy). Samples with detectable hemolysis were excluded from the analysis.

Organs and blood sampling

At of sacrifice, tissues were removed, dissected, snap-frozen in liquid nitrogen, and stored at -80°C until use. For the ileum section, the isolation of single cells was performed by a modification of the Weiser method, as previously described [3, 4]. Briefly, the ileum was harvested, washed with a cold saline solution, and cut longitudinally. To remove the luminal content, the ileum was transferred into Hank's balanced salt solution with 0.5 mM DTT. Then, ileum was placed into 20 ml of Chelating Buffer (Na_2PO_4 5mM, NaCl 96mm, Na Citrate27mM, KH_2PO_4 8mM, KCl 1.5 mm, D-Sorbitol 55mM, Sucrose 44mM, DTT 0.5 mM), and incubated at 4˚C with constant stirring for 20 minutes and the supernatant was collected. After, the ileum was transferred into a 50ml tube with 15 ml of Chelating buffer, gently shaken by hand (30 inversions) and the supernatant was collected. This operation was repeated eight times. All collected supernatants were centrifuged at 1000g, washed with cold saline buffer, and then stored at -80°C. Blood was collected by cardiac puncture using heparin-coated syringes. Plasma was prepared by centrifugation (1000×g, 10 min at 4°C) and kept at -80°C until use.

Measurement of Endogenous Respiratory Fluxes in Intact Enterocytes

Respiration rates were measured polarographically with a Clark-type oxygen electrode in a water-jacketed chamber (Hansatech Instruments), magnetically stirred at 37 °C as previously described [3]. Briefly, pooled fractions of freshly isolated enterocytes were collected by centrifugation and then were transferred into the polarographic chamber at 1–2 mg of protein/mL in TD buffer [0.137 M NaCl, 5 mM KCl, 0.7 mM Na_2HPO_4, 25 mM Tris·HCl (pH 7.4)] air-equilibrated at 37 °C. The respiration rate by endogenous substrates was read directly (endogenous respiration) and after the addition of 60 µM 2,4-dinitrophenol

(uncoupled respiration). The in-situ cytochrome c oxidase activity (A-T) was measured as the KCN-sensitive oxygen consumption rate elicited by 10 mM sodium ascorbate + 0.4 mM N,N,N′,N′,-tetramethyl-phenylenediamine in the presence of antimycin A.

Gene Expression

For the liver, ileum single cells, and gastrocnemius, total RNA was extracted with Qiazol reagent (Qiagen, Germany). For inguinal white adipose tissue, total RNA was extracted using the RNeasy Lipid Tissue Mini Kit (Qiagen, Germany), following the instructions. 1-2 µg of total RNA were treated with DNase (Thermo Fisher Scientific, USA) and retrotranscribed to cDNA using the High Capacity cDNA Reverse Transcription Kit (Thermo Fisher Scientific, Massachusetts, USA) following the manufacturer’s instructions. qPCR assays were performed in 96-well plates using the Master Mix Power SYBR Green (Thermo Fisher Scientific, USA) via the QuantStudio5 machine (Thermo Fisher Scientific, USA), and the first analysis was performed using the QuantStudio Design & Analysis. Relative quantification was calculated via the ΔΔCT method, using Tbp (TATA-binding protein) as a reference gene. All the primers used in this study are listed in Supplementary Table 1.

Transcriptome profiles were obtained for 6 intestinal samples per group at the GeT-TRiX facility (GénoToul, Génopole Toulouse Midi-Pyrénées) using Sureprint G3 Mouse GE v2 microarrays (8 3 60K, design 074809, Agilent Technologies), according to the manufacturer’s instructions. For each sample, Cyanine-3 (Cy3) labelled cRNA was prepared from 200 ng of total RNA using the One-Color Quick Amp Labeling kit (Agilent Technologies), according to the manufacturer’s instructions, followed by Agencourt RNAClean XP (Agencourt Bioscience Corporation, Beverly, Massachusetts). Dye incorporation and cRNA yield were determined using a Dropsense 96 UV/VIS droplet reader (Trinean, Belgium). Next, 600 ng of Cy3-labeled cRNA were hybridized on the microarray slides, following the manufacturer’s instructions. Immediately after washing, slides were scanned on an Agilent G2505C Microarray Scanner using Agilent Scan Control A.8.5.1 software, and the fluorescence signal was extracted using Agilent Feature Extraction software v10.10.1.1 with default parameters. Microarray data and experimental details are available in NCBI’s Gene Expression Omnibus (GEO) database (accession numbers GSE227610).

Publicly available mRNA expression profiles were obtained by gene expression omnibus (GEO Accession Number: GSE113819, www.ncbi.nlm.nih.gov/geo/query/acc.cgi?acc=GSE113819). Specifically, since the search terms “NAFLD” OR “NASH” OR “liver steatosis” OR “steatohepatitis” AND “small intestine” AND “homo sapiens” do not retrieve any information, we used the search terms “obesity”

AND “small intestine” AND “homo sapiens”, recovering 7 different datasets. Datasets with specimens from other organisms, expression profiles by RT-qPCR or RNA sequencing, or the absence of a clear control group were excluded.

Microarray Analysis

Microarray data were analyzed using R and Bioconductor packages [5]. Raw data (median signal intensity) were filtered, log2 transformed, corrected for batch effects (microarray washing bath and labelling serials), and normalized using the qsmooth method [6]. A model was fitted using the limma lmFit function [7]. Pairwise comparisons between biological conditions were applied using specific contrasts. A correction for multiple testing was applied using the Benjamini-Hochberg procedure to control the false discovery rate (FDR). Probes with an FDR≤0.05 were considered to be differentially expressed between conditions. Hierarchical clustering was applied to the samples and the differentially expressed probes using 1-Pearson correlation coefficient as distance and Ward’s criterion for agglomeration. The clustering results are illustrated as a heatmap of expression signals. Gene ontology and transcription factor enrichment analysis were performed using Metascape [8].

Histology and Immunohistochemistry

Tissue specimens were fixed in 10% formalin for 12-24 hours, dehydrated, and paraffin-embedded. Ileum and liver sections (2 µm) were stained with hematoxylin-eosin staining (HE), according to the standard procedures. Villi length was calculated by evaluating complete, full-sized intestinal villi (n=10) not exhibiting bending or mechanical damage, for each sample. Sirius Red staining using Direct Red 80 and Fast Green FCF (Sigma-Aldrich, USA) was performed on liver sections to assess fibrosis. Immunohistochemistry analysis was performed in liver and ileum specimens (4 µm). Briefly, sections were subjected to antigen retrieval by boiling the slides in sodium citrate pH 6 for 15 minutes, permeabilized in phosphate-buffered saline with 0.25% Triton X-100 for 5 minutes, and then incubated for 10 minutes at room temperature in protein blocking solution (Dako, Denmark). Subsequently, sections were incubated with primary antibodies as indicated in Supplementary Table 2. Sections were washed in PBS for 15 minutes and incubated at room temperature with DAKO real EnVision detection system (Dako, Denmark), according to the manufacturer’s instruction. For negative controls, 1% nonimmune serum in PBS substituted the primary antibodies. Images were acquired and analyzed with Aperio Image Scope (Leica Biosystems, Germany). The percentage of stained area/total area was evaluated in 10 consecutive acquired images. Values from all consecutive images for each sample were

averaged and displayed as mean±SEM. Steatosis score was assigned based on the percentage of hepatic parenchyma containing fat: 0 - <5%; 1 – 5-33%; 2 – 33-66%; 3 - >66% [9].

Specimens from livers were embedded in OCT (Sakura), frozen under nitrogen vapours, and stored at –80°C. Liver cryosections (4 µm) were stained with Oil Red O Stain Kit (ab150678, Abcam, UK) following the manufacturer's indications.

Lipidomic Assay

Fatty acids were extracted from frozen tissues or plasma using a modified Bligh and Dyer extraction method. Samples were lysed in a water EDTA (5 Mm)/methanol mix (1:2 vol/vol). Methanol and dichloromethane were added to reach the following ratios of MeOH/water/CH2Cl2: 2.5/2.0/2.5. Glyceryltrinonadecanoate was added as an internal standard. The dried lipid extract was transmethylated with 1 ml of BF3 in methanol (1:20, vol/vol) for 60 min at 100°C, evaporated to dryness, and the fatty acid methyl esters (FAMEs) were extracted with hexane/water (3:1). The organic phase was evaporated to dryness and dissolved in 50 µl ethyl acetate. FAMEs were analyzed by gas-liquid chromatography on a 5890 Hewlett-Packard system using a Famewax fused-silica capillary column (30 m, 0.32 mm i.d., 0.25-mm film thickness; Restek). The oven temperature was programmed from 110ºC to 220°C at a rate of 2°C/min, and the carrier gas was hydrogen (0.5 bar). The injector and the detector were at 225ºC and 245°C, respectively. Neutral lipids were extracted from plasma, liver or intestine frozen tissues using a Bligh and Dyer extraction method: samples were homogenized in methanol/5 mM EGTA (2:1, v/v), and lipids (corresponding to an equivalent of 2mg tissue) extracted according to the Bligh–Dyer method63, with chloroform/methanol/water (2.5:2.5:2 v/v/v), in the presence of the following internal standards: glyceryl trinonadecanoate, stigmasterol, and cholesteryl heptadecanoate (Sigma-Aldrich). Triglycerides, free cholesterol, and cholesterol esters were analyzed by gas-liquid chromatography on a Focus Termo Electron system equipped with a Zebron- 1 Phenomenex fused-silica capillary column (5 m, 0.25 mm i.d., 0.25 mm film thickness). The oven temperature was programmed to increase from 200 to 350 °C at 5 °C/min, and the carrier gas was hydrogen (0.5 bar). The injector and detector temperatures were 315 °C and 345 °C, respectively.

Statistical Analysis

All the results are expressed as mean ± SEM. Statistical analyses were performed with GraphPad Prism software analysis (v9.0, GraphPad Software, USA). Outliers were

calculated with ROUT or Grubbs test. To compare two groups Mann Whitney U test was used, while for four groups 2-Way ANOVA followed by Tukey's post-hoc test. A p-value <0.05 was considered significant. Paired T-Test was used to analyze paired data. Significant genotype effect was indicated by * (*$p < 0.05$, **$p < 0.01$, ***$p < 0.001$). Significant diet effect was indicated by $^{\#}$ ($^{\#}p < 0.05$, $^{\#\#}p < 0.01$, $^{\#\#\#}p < 0.001$).

Supplementary Figure 1

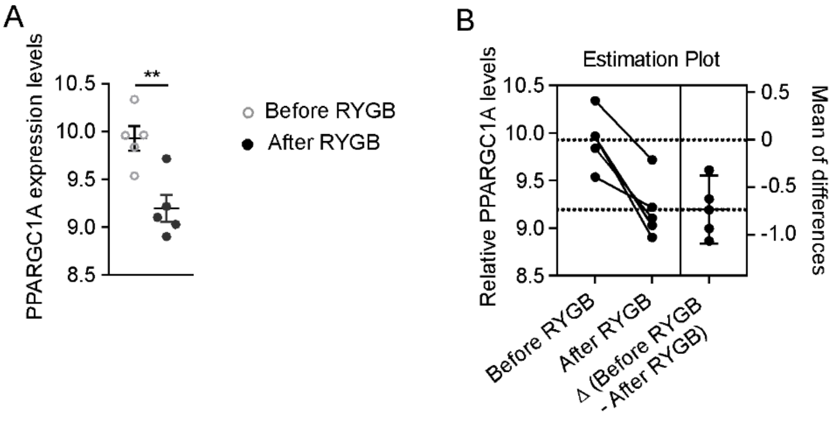

Supplementary Figure 1. PPARGC1A expression is increased in obese individuals. (A) PPARGC1A expression levels in small intestine harvested from obese patients before and after RYGB bypass (GSE113819) and (B) estimation plot of the PPARGC1A expression: on the left, scatter plot showing individual points; on the right, the effect size (difference between means). Data are expressed as mean±SEM. Comparison between distinct groups was performed using Paired T-test (**p<0.01).

Supplementary Figure 2

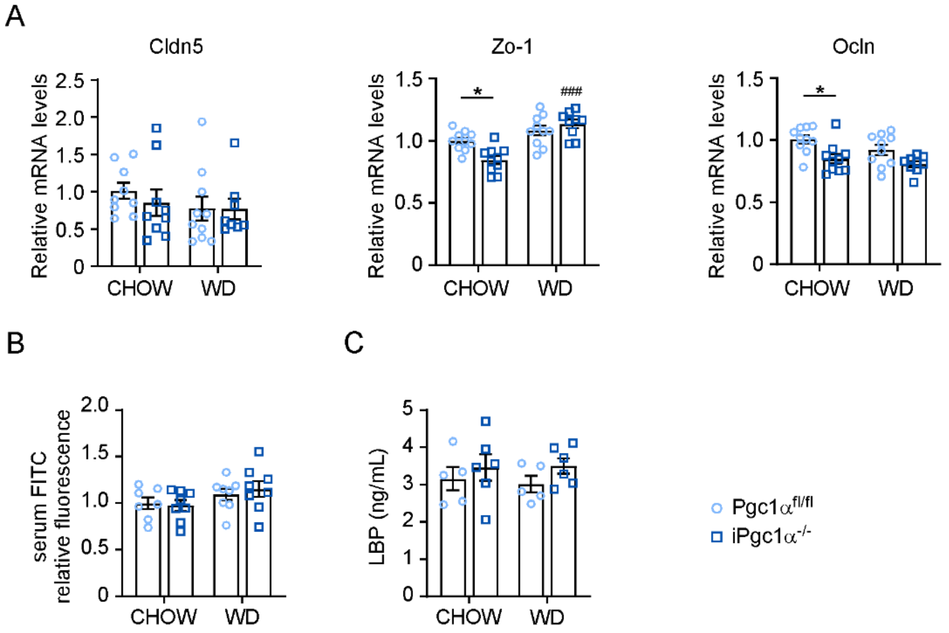

Supplementary Figure 2. Pgc1α ablation does not impair intestinal permeability. (A) Relative mRNA expression of tight junction proteins, Claudin 2 (Cldn5), Zona Occludens 1 (Zo-1), and Occludin (Ocln) measured in the ileum. (B) *In vivo* intestinal permeability measured by FITC assay. (E) Lipopolysaccharide binding protein measured in plasma. All the experiments were performed on 2 months old iPgc1α$^{-/-}$ and Pgc1α$^{fl/fl}$ littermates fed with chow or western diet (WD) for 2 months. (n=6-10 animals/group). Data are expressed as mean±SEM. Comparison between distinct groups was performed using Two-way ANOVA followed by Sidak's multiple comparison test; *genotype effect $^{\#}$diet effect (* or $^{\#}$p<0.05; ** or $^{\#\#}$p<0.01; *** or $^{\#\#\#}$p<0.001).

Supplementary Figure 3

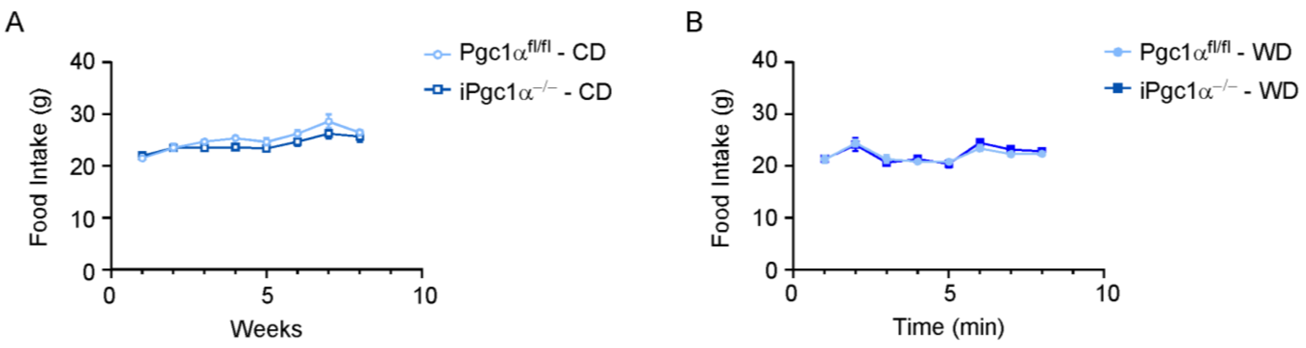

Supplementary Figure 3. Intestinal Pgc1α ablation does not alter food intake. Food intake of iPgc1α$^{-/-}$ mice and their Pgc1α$^{fl/fl}$ littermates fed (A) chow diet and (B) western diet (WD) for 2 months. All the experiments were performed iPgc1α$^{-/-}$ and littermates' control Pgc1α$^{fl/fl}$ (n=10 animals/group). Data are expressed as mean±SEM. Comparison between two groups was performed using Multiple Mann Whitney Test.

Supplementary Figure 4

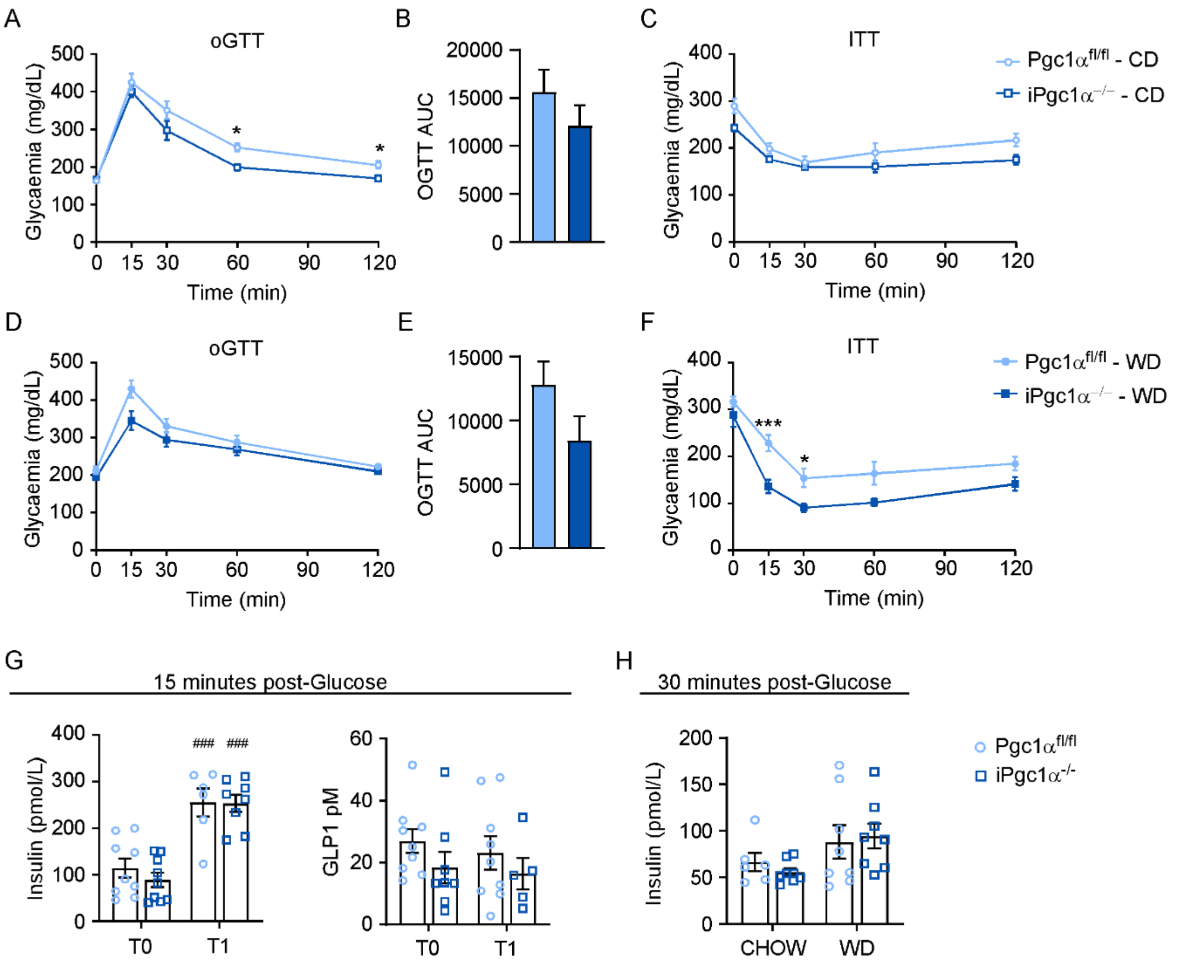

Supplementary Figure 4. Intestinal Pgc1α ablation does not alter glucose tolerance. (A) Oral glucose tolerance test, (B) relative Area Under the Curve (AUC), and (C) insulin tolerance test performed in fasted chow diet fed animals. (D) Oral glucose tolerance test, (E) relative AUC, and (F) insulin tolerance test performed in fasted western diet fed animals. (G) Serum insulin and GLP1 levels at basal level and 15 minutes after an intragastric bolus of glucose in fasted chow diet fed animals. (H) Serum insulin levels 30 minutes after an intragastric bolus of glucose in fasted chow diet and western fed animals. All the experiments were performed on 2 months old iPgc1α$^{-/-}$ mice and their Pgc1α$^{fl/fl}$ littermates were fed with chow or western diet (WD) (n=10 animals/group). Data are expressed as mean±SEM. Comparison between distinct groups was performed using Two-way ANOVA followed by Sidak's multiple comparison test; *genotype effect $^{\#}$diet effect (* or $^{\#}$p<0.05; *** or $^{\#\#\#}$p<0.001).

Supplementary Figure 5

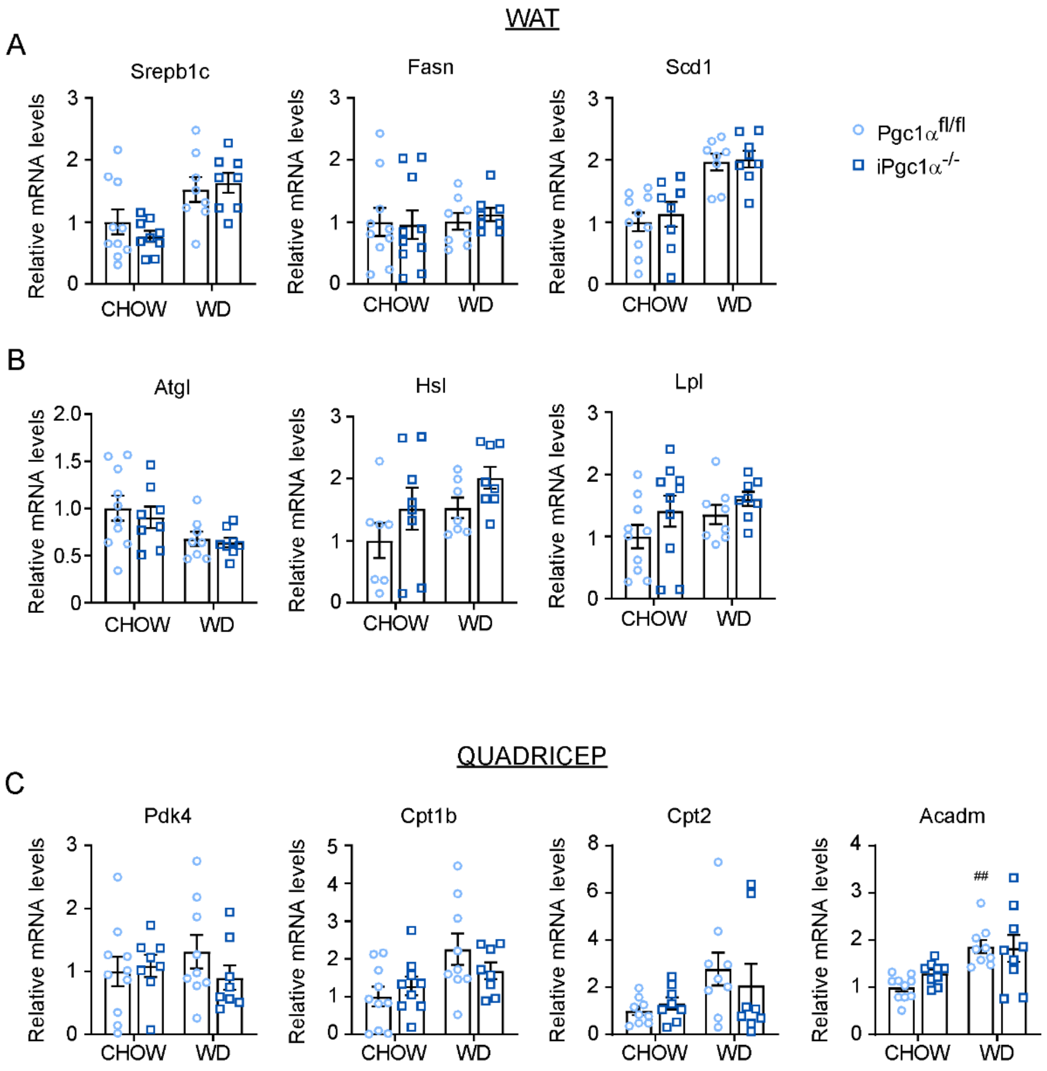

Supplementary Figure 5. Intestinal Pgc1α ablation does not modulate white adipose tissue and muscle gene expression. (A) Relative mRNA expression of genes involved in de novo lipogenesis in the WAT. (B) Relative mRNA expression of genes involved in lipolysis in the WAT. (C) Relative mRNA expression of genes involved in fatty acids β-oxidation in the quadriceps. All the experiments were performed on 2 months old iPgc1α$^{-/-}$ mice and their Pgc1α$^{fl/fl}$ littermates were fed with chow or western diet (WD) (n=10 animals/group). Data are expressed as mean±SEM. Comparison between distinct groups was performed using Two-way ANOVA followed by Sidak's multiple comparison test.

Supplementary Table 1
List of primers used in the study.

Gene	Sequence Primer Forward	Sequence Primer Reverse
Abcg5	TCAATGAGTTTTACGGCCTGAA	GCACATCGGGTGATTTAGCA
Abcg8	AATGTCATCCTGGATGTCGTCTC	CCAGCTCATAGTACAGCATTGACC
Acadm	GCAGGGTCCTGAGAAGTGTTC	GCTTGTGAGCCGCTTTCG
Acta2	GTTCAGTGGTGCCTCTGTCA	ACTGGGACGACATGGAAAAG
Arg1	AACACTCCCCTGACAACCAG	CCAGCAGGTAGCTGAAGGTC
Atgl	GCAACCTGTCATTGTTCTAGC	CCAACGCCACTCACATCTAC
Atp5b	CACCAAGAAGGGATCGATCAC	GCAGGGTCAGTCAGGTCATCA
Ccl2	CCTTTTCCACAACCACCTCAAG	TAATTAAGGCATCACAGTCCGAGTC
Cd206	ATCCACTCTATCCACCTTCA	TGCTTGTTCATATCTGTCTTCA
Cd68	CTTCCCACAGGCAGCACAG	TGTAGCCTTAGAGAGAGCAGGTCA
Cldn5	GTGGAACGCTCAGATTTCAT	TGGACATTAAGGCAGCATCT
Col1a1	TAGGCCATTGTGTATGCAGC	ACATGTTCAGCTTTGTGGACC
Cpt1a	GAAGAAGAAGTTCATCCGATTCAAG	GATATCACACCCACCACCACG
Cpt1b	AGGGCTGCACTCCTGGAAG	CACGATAAGCCAGCTGGAGG
Cpt2	ACCATGCACTACCAGGACAGC	TGTCTTCAAGTTTAGGGATAGGCA
CytC	GGTGATGTTGAAAAAGGCAAGAA	TTGCCTCCCTTTTCCACAGT
Fabp1	CCCTTGATGTCCTTCCCTTTC	TTGCCACCATGAACTTCTCC
Fat/Cd36	GATGACGTGGCAAAGAACAG	TCCTCGGGGTCCTGAGTTAT
Fatp4	TGAAATCACCGCAGACGACAGG	GCTTGTCACCATCTCGTTTTCTC
Fasn	AGTCAGCTATGAAGCAATTGTGGA	CACCCAGACGCCAGTGTTC
Hmgcr	CTTGTGGAATGCCTTGTGATTG	AGCCGAAGCAGCACATGAT
Hsl	GCTGGGCTGTCAAGCACTGT	GTAACTGGGTAGGCTGCCAT
Il1b	CCTCAATGGACAGAATATCAACCAA	TCTCCTTGTACAAAGCTCATGGAG
Ldlr	AGGCTGTGGGCTCCATAGG	TGCGGTCCAGGGTCATCT
Lpl	AGCAGACGCGGGAAGAGA	AAGGTCTTGCTGCTGTGGTTG
Lxrα	AGGAGTGTCGACTTCGCAAA	CTCTTCTTGCCGCTTCAGTTT
Lxrβ	AAGCAGGTGCCAGGGTTCT	TGCATTCTGTCTCGTGGTTGT
Mmp9	CTCGAGGGCTTCCCTCTGA	GGCTGGAGGCCTTGGGT
Mmp13	AGGCCTTCAGAAAAGCCTTC	TCCTTGGAGTGATCCAGACC
Mttp	TCAGGAAGCTGTGTCAGAATGAAG	TTTCAAGTCCTCCCAGGATCA
Npc1l1	AGATCCCAACTTTGAGGTCTTCC	ACCGTCAGGTATTGCTGGTAGAAC
Ocln	TTGAAGAGTGGGTTAAAAATGTGTCT	TCAACTCTTTCCGCATAGTCAGAT
Pdk4	CCAGGGAGGTCGAGCTGTT	TGTCAATCTCCTTCAGGATATTGG
Pgc1α	TTGATGCACTGACAGATGGAG	GCTGAGTGTTGGCTGGT
Scarb1	TCCCCATGAACTGTTCTGTGAA	TGCCCGATGCCCTTGA
Scd1	CAGTGCCGCGCATCTCTAT	CAGCGGTACTCACTGGCAGA
Srebp1c	GGAGCCATGGATTGCACATT	GGCCCGGGAAGTCACTGT
Tfam	GGTCGCATCCCCTCGTCTA	GGATAGCTACCCATGCTGGAAA
Tgfb	GCAGTGGCTGAACCAAGGA	AGAGCAGTGAGCGCTGAATC
Tnfa	CTGAGGTCAATCTGCCCAAGTAC	CTTCACACAGCAATGACTCCAAAG
Zo-1	AGGACACCAAAGCATGTGAG	GGCATTCCTGCTGGTTACA

Supplementary Table 2
List of antibodies used in the study.

Antibody	Company	Catalogue	Host	Dilution
Pgc1α	Abcam	LS-C415095	Rabbit	1:1000
F4/80	Cell Signaling	D2S9R	Rabbit	1:200

Supplementary References

[1] Moschetta A, Frederik PM, Portincasa P, vanBerge-Henegouwen GP, van Erpecum KJ. The incorporation of cholesterol in sphingomyelin- phosphatidylcholine vesicles has profound effects on detergent-induced phase transitions. J Lipid Res 2002;43:1046-1053.

[2] Lopez CA, de Vries AH, Marrink SJ. Molecular mechanism of cyclodextrin mediated cholesterol extraction. PLoS Comput Biol 2011;7:e1002020.

[3] Bellafante E, Morgano A, Salvatore L, Murzilli S, Di Tullio G, D'Orazio A, et al. PGC-1beta promotes enterocyte lifespan and tumorigenesis in the intestine. Proc Natl Acad Sci U S A 2014;111:E4523-4531.

[4] Contursi A, Arconzo M, Cariello M, Piglionica M, D'Amore S, Vacca M, et al. let-7e downregulation characterizes early phase colonic adenoma in APCMin/+ mice and human FAP subjects. PLoS One 2021;16:e0249238.

[5] Huber W, Carey VJ, Gentleman R, Anders S, Carlson M, Carvalho BS, et al. Orchestrating high-throughput genomic analysis with Bioconductor. Nat Methods 2015;12:115-121.

[6] Hicks SC, Okrah K, Paulson JN, Quackenbush J, Irizarry RA, Bravo HC. Smooth quantile normalization. Biostatistics 2018;19:185-198.

[7] Ritchie ME, Phipson B, Wu D, Hu Y, Law CW, Shi W, et al. limma powers differential expression analyses for RNA-sequencing and microarray studies. Nucleic Acids Res 2015;43:e47.

[8] Zhou Y, Zhou B, Pache L, Chang M, Khodabakhshi AH, Tanaseichuk O, et al. Metascape provides a biologist-oriented resource for the analysis of systems-level datasets. Nat Commun 2019;10:1523.

[9] Kleiner DE, Brunt EM, Van Natta M, Behling C, Contos MJ, Cummings OW, et al. Design and validation of a histological scoring system for nonalcoholic fatty liver disease. Hepatology 2005;41:1313-1321.

JOURNAL
OF HEPATOLOGY

Journal of Hepatology

CTAT methods

Tables for a "Complete, Transparent, Accurate and Timely account" (CTAT) are now mandatory for all revised submissions. The aim is to enhance the reproducibility of methods.

- Only include the parts relevant to your study
- Refer to the CTAT in the main text as 'Supplementary CTAT Table'
- Do not add subheadings
- Add as many rows as needed to include all information
- Only include one item per row

1.1 Antibodies

Name	Citation	Supplier	Cat no.	Clone no.
Pgc1α		Abcam	ab191838	Polyclonal
F4/80		Cell Signaling	#70076	D2S9R

1.2 Organisms

Name	Citation	Supplier	Strain	Sex	Age	Overall n number
Wild type	-	Jackson Laboratory	C57BL6/J	Male	8 weeks	40
Pgc1α$^{fl/fl}$	PMID: 22087241	Anastasia Kralli	C57BL6/J	Male	8 weeks	100
Vil1-Cre	-	Jackson Laboratory	C57BL6/J	Female	8 weeks	2
iPgc1α$^{-/-}$	-	-	C57BL6/J	Male	8 weeks	100

1.3 Deposited data

Name of repository	Identifier	Link
Gene Expression Omnibus	GSE227610	www.ncbi.nlm.nih.gov/geo
Gene Expression Omnibus	GSE113819	www.ncbi.nlm.nih.gov/geo

1.4 Software

Software name	Manufacturer	Version
GraphPad Prism	GraphPad Software	v9.0
QuantStudio Design & Analysis	Applied Biosystems	V1.3.1
RStudio	RStudio, PBC	2022.07.2+576
Aperio Image Scope	Leica Biosystems	12.4.3.5008

1.5 Please provide the details of the corresponding methods author for the manuscript:

antonio.moschetta@uniba.it
