# Supplementary material for: Intestinal Pgc1α ablation protects from liver steatosis and fibrosis
Source: JHEP Rep. 2023 Jul 19;5(11):100853. doi: 10.1016/j.jhepr.2023.100853 (PMC10597770; doi:10.1016/j.jhepr.2023.100853)
Supplement: Multimedia component 2 [file mmc2.docx]

**Journal of Hepatology**

**CTAT methods**

Tables for a “Complete, Transparent, Accurate and Timely account” (CTAT) are now mandatory for all revised submissions. The aim is to enhance the reproducibility of methods.

- Only include the parts relevant to your study
- Refer to the CTAT in the main text as ‘Supplementary CTAT Table’
- Do not add subheadings
- Add as many rows as needed to include all information
- Only include one item per row
  1. **Antibodies**

| **Name** | **Citation** | **Supplier** | **Cat no.** | **Clone no.** |
| --- | --- | --- | --- | --- |
| Pgc1α |  | Abcam | ab191838 | Polyclonal |
| F4/80 |  | Cell Signaling | #70076 | D2S9R |

- 1. **Organisms**

| **Name** | **Citation** | **Supplier** | **Strain** | **Sex** | **Age** | **Overall n number** |
| --- | --- | --- | --- | --- | --- | --- |
| Wild type | - | Jackson Laboratory | C57BL6/J | Male | 8 weeks | 40 |
| Pgc1α^fl/fl^ | PMID: 22087241 | Anastasia Krallli | C57BL6/J | Male | 8 weeks | 100 |
| Vil1-Cre | - | Jackson Laboratory | C57BL6/J | Female | 8 weeks | 2 |
| iPgc1α^-/-^ | - | - | C57BL6/J | Male | 8 weeks | 100 |

- 1. **Deposited data**

| **Name of repository** | **Identifier** | **Link** |
| --- | --- | --- |
| Gene Expression Omnibus | GSE227610 | www.ncbi.nlm.nih.gov/geo |
| Gene Expression Omnibus | GSE113819 | www.ncbi.nlm.nih.gov/geo |

- 1. **Software**

| **Software name** | **Manufacturer** | **Version** |
| --- | --- | --- |
| GraphPad Prism | GraphPad Software | v9.0 |
| QuantStudio Design & Analysis | Applied Biosystems | V1.3.1 |
| RStudio | RStudio, PBC | 2022.07.2+576 |
| Aperio Image Scope | Leica Biosystems | 12.4.3.5008 |

- 1. **Please provide the details of the corresponding methods author for the manuscript:**

| antonio.moschetta@uniba.it |
| --- |
