# Supplementary material for: Intestinal Pgc1α ablation protects from liver steatosis and fibrosis
Source: JHEP Rep. 2023 Jul 19;5(11):100853. doi: 10.1016/j.jhepr.2023.100853 (PMC10597770; doi:10.1016/j.jhepr.2023.100853)
Supplement: Multimedia component 4 [file mmc4.pdf]

ARTICLE IN PRESS

Research article

JHEP|Reports

Intestinal Pgc1α ablation protects from liver steatosis and fibrosis

Authors

Elena Piccinin, Maria Arconzo, Maria Laura Matrella, Marica Cariello, Arnaud Polizzi, Yannick Lippi, Justine Bertrand-Michel, Hervé Guillou, Nicolas Loiseau, Gaetano Villani, Antonio Moschetta

Correspondence

antonio.moschetta@uniba.it (A. Moschetta).

Graphical abstract

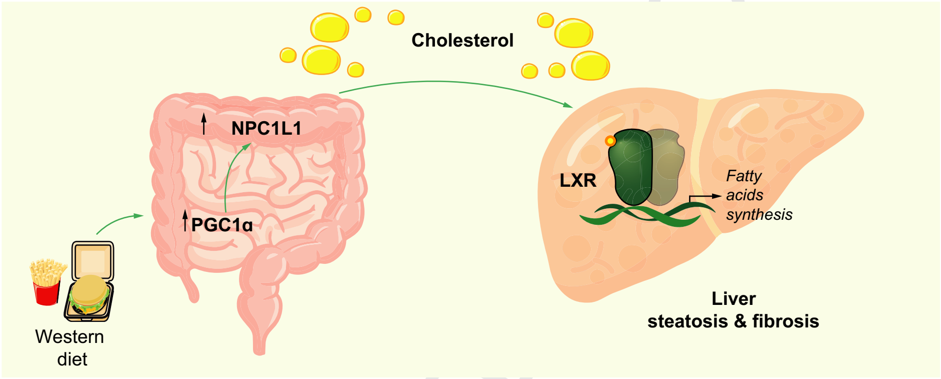

Highlights

- Metabolic liver diseases may be caused by altered gut–liver crosstalk.
- Cholesterol represents a deleterious lipotoxic species, involved in steatohepatitis.
- Pgc1α controls intestinal cholesterol uptake by regulating the expression of the cholesterol transporter Npc1l1.
- Intestinal Pgc1α deletion protects the liver from lipid accumulation, inflammation, and fibrosis.

Impact and implications

Liver diseases result from several insults, including signals from the gut. Although the incidence of liver diseases is continuously increasing worldwide, effective drug therapy is still lacking. Here, we showed that the modulation of an intestinal coactivator regulates the liver response to a Western diet, by limiting the uptake of dietary cholesterol. This results in a lower accumulation of hepatic lipids together with decreased inflammation and fibrosis, thus limiting the progression of liver steatosis and fibrosis towards severe end-stage diseases.

UNCORRECTED PROOF

https://doi.org/10.1016/j.jhepr.2023.100853

UNCORRECTED PROOF

FLA 5.6.0 DTD ■ JHEPR100853_grabs ■ 14 September 2023 ■ 8:07 pm ■ ce

Intestinal Pgc1α ablation protects from liver steatosis and fibrosis

Elena Piccinin,[1,2] Maria Arconzo,[1] Maria Laura Matrella,[2] Marica Cariello,[1] Arnaud Polizzi,[3] Yannick Lippi,[3] Justine Bertrand-Michel,[4] Hervé Guillou,[3] Nicolas Loiseau,[3] Gaetano Villani,[2] Antonio Moschetta[1,5,*]

[1]*Department of Interdisciplinary Medicine, University of Bari 'Aldo Moro', Bari, Italy;* [2]*Department of Translational Biomedicine and Neuroscience (DiBraiN), University of Bari 'Aldo Moro', Bari, Italy;* [3]*Toxalim (Research Center in Food Toxicology), INRAE, ENVT, INP-PURPAN, UMR 1331, UPS, Université de Toulouse, Toulouse, France;* [4]*MetaboHUB-MetaToul, National Infrastructure of Metabolomics and Fluxomics, Toulouse, France;* [5]*INBB, National Institute for Biostructures and Biosystems, Rome, Italy*

JHEP Reports **2023.** https://doi.org/10.1016/j.jhepr.2023.100853

Background & Aims: The gut–liver axis modulates the progression of metabolic dysfunction-associated steatotic liver disease (MASLD), a spectrum of conditions characterised by hepatic steatosis and a progressive increase of inflammation and fibrosis, culminating in metabolic dysfunction-associated steatohepatitis. Peroxisome proliferator-activated receptor-gamma coactivator 1α (Pgc1α) is a transcriptional co-regulator of mitochondrial activity and lipid metabolism. Here, the intestinal-specific role of Pgc1α was analysed in liver steatosis and fibrosis.
Methods: We used a mouse model in which Pgc1α was selectively deleted from the intestinal epithelium. We fed these mice and their wild-type littermates a Western diet to recapitulate the major features of liver steatosis (after 2 months of diet) and metabolic dysfunction-associated steatohepatitis (after 4 months of diet). The chow diet was administered as a control diet.
Results: In humans and mice, low expression of intestinal Pgc1α is inversely associated with liver steatosis, inflammation, and fibrosis. Intestinal disruption of Pgc1α impairs the transcription of a wide number of genes, including the cholesterol transporter Niemann–Pick C1-like 1 (*Npc1l1*), thus limiting the uptake of cholesterol from the gut. This results in a lower cholesterol accretion in the liver and a decreased production of new fatty acids, which protect the liver from lipotoxic lipid species accumulation, inflammation, and related fibrotic processes.
Conclusions: In humans and mice, intestinal Pgc1α induction during Western diet may be another culprit driving hepatic steatosis and fibrosis. Here, we show that enterocyte-specific Pgc1α ablation protects the liver from steatosis and fibrosis by reducing intestinal cholesterol absorption, with subsequent decrease of cholesterol and *de novo* fatty acid accumulation in the liver.
Impact and implications: Liver diseases result from several insults, including signals from the gut. Although the incidence of liver diseases is continuously increasing worldwide, effective drug therapy is still lacking. Here, we showed that the modulation of an intestinal coactivator regulates the liver response to a Western diet, by limiting the uptake of dietary cholesterol. This results in a lower accumulation of hepatic lipids together with decreased inflammation and fibrosis, thus limiting the progression of liver steatosis and fibrosis towards severe end-stage diseases.
© 2023 The Author(s). Published by Elsevier B.V. on behalf of European Association for the Study of the Liver (EASL). This is an open access article under the CC BY license (http://creativecommons.org/licenses/by/4.0/).

Introduction

Metabolic dysfunction-associated steatotic liver disease (MASLD), formerly termed non-alcoholic fatty liver disease (NAFLD), represents the world's leading cause of chronic liver disease, with more than 25% of the global population affected.[1] This number is proportionally increasing with the raising of metabolic syndrome, obesity, insulin resistance, and diabetes mellitus type 2. Hepatic fat accumulation is the sign of MASLD, which may progressively lead to liver dysfunctions, inflammatory cell infiltration, and scarring peculiar to metabolic dysfunction-associated steatohepatitis (MASH), formerly termed non-alcoholic steatohepatitis (NASH), with a crescendo towards advanced liver diseases, cirrhosis, and hepatocellular carcinoma. However, despite the efforts made to characterise these diseases, MASLD and its sequelae remain without effective drug treatment. Bariatric surgery often remains the last therapeutic option for individuals with morbid obesity and MASH.[2]

Hepatic steatosis is a well-recognised hallmark of MASLD, and it is caused by the build-up of different lipid species in the liver, including triglycerides and cholesterol. An excess of triglycerides may derive from increased adipose tissue lipolysis, *de novo* lipogenesis, or a nutritional overload. However, although triglycerides are the most abundant hepatic lipid species, they

Keywords: Metabolic dysfunction-associated steatotic liver disease; Metabolic dysfunction-associated steatohepatitis; Gut–liver axis; Cholesterol; Peroxisome proliferator-activated receptor-gamma coactivator 1α; NAFLD; non-alcoholic fatty liver disease; NASH; non-alcoholic steatohepatits.
Received 12 April 2023; received in revised form 4 July 2023; accepted 7 July 2023; available online 19 July 2023
* Corresponding author. Address: Department of Interdisciplinary Medicine, University of Bari 'Aldo Moro', Piazza Giulio Cesare 11, 70124, Bari, Italy. Tel.: +390805593262
E-mail address: antonio.moschetta@uniba.it (A. Moschetta).

represent a 'safe' storage solution in the liver.[3] By contrast, an overabundance of cholesterol has deleterious hepatic effects, culminating with the so-called cholesterol-associated steatohepatitis (CASH).[3] When the synthesis or uptake of cholesterol is increased and/or the cholesterol excretion is reduced, cholesterol starts to accumulate within the hepatocyte's lipid droplets. By inhibiting the circulating proprotein convertase subtilisin/kexin type 9 (PCSK9), an increased expression of the LDL receptor (LDLR) that facilitates the LDL uptake occurs.[4] This exposes the liver to a considerable amount of cholesterol that, on one side starts to crystalise within hepatocyte lipid droplets, driving necroinflammation and liver dysfunctions,[4] and on the other, blocks the proteasomal degradation of TAZ, promoting fibrosis.[5] Overall, this results in a higher risk of steatohepatitis and liver cancer.

Recent investigations have postulated a crucial role of the gut–liver axis in the promotion of metabolic liver diseases.[6] The gut participates in liver lipotoxicity through a wide range of signals, including the metabolism of nutrients, the release of secretory molecules, and microbiota modifications.[6–8]

Peroxisome proliferator-activated receptor gamma coactivator 1α (Pgc1α) was first described as a coactivator involved in the promotion of metabolic pathways especially under conditions of energy deprivation. Mostly expressed in highly metabolic organs, Pgc1α regulates the expression of genes involved in mitochondrial metabolism, antioxidant response, gluconeogenesis, and fatty acid β-oxidation.[9] In the gut, Pgc1α regulates the apoptotic processes that physiologically take place at the tip of the villi.[10,11] In *Drosophila melanogaster*, the intestinal overexpression of the Pgc1α homologue is essential to modulate gut permeability, maintaining homoeostasis and prolonging the lifespan.[12] In line with this, disruption of Pgc1α expression has been associated with colitis and colorectal cancer in both humans and mice.[11,13] However, whether the ablation of Pgc1α in the intestine can promote liver steatosis and fibrosis onset has not been investigated so far.

Here, we take advantage of an engineered mouse model to explore whether intestinal Pgc1α is crucial in the development of hepatic steatosis and fibrosis. Surprisingly, we found that the lack of Pgc1α from the gut impairs cholesterol absorption, finally antagonising the onset of liver steatosis and the progression to fibrosis.

Materials and methods

Animals

Mice were kept in a pathogen-free facility, at 21 ± 2 °C with a 12-h light/dark cycle, and had free access to food and water. All the murine strains we used were in C57BL6/J background. To generate iPgc1α$^{-/-}$ mice, Pgc1α$^{fl/fl}$ mice[14] were intercrossed with Vil1-Cre mice (Jackson Laboratory, Bar Harbor, ME, USA) to obtain Vil1-Cre$^{Tg/-}$Pgc1α$^{fl/-}$ mice. These mice were then backcrossed with Pgc1α$^{fl/fl}$ mice to restore homozygosity for the iPgc1α floxed allele. Male Vil1-Cre$^{Tg/-}$Pgc1α$^{fl/fl}$ mice were bred to Pgc1α$^{fl/fl}$ mice to produce the mice used in the study. Pgc1α$^{fl/fl}$ mice were used as controls. Eight-week-old mice were treated for 8 or 16 weeks with 42% kcal/fat diet enriched with saturated fats (0.2% total cholesterol, milk fat) (Teklad, TD.180342). Chow diet was used as a control. Mice were randomly assigned to treatment groups for *in vivo* studies. Food intake and body weight were monitored weekly. All mice were sacrificed randomly after overnight fasting at Zeitgeber Time (ZT) 3. Each animal experiment was repeated using at least two cohorts of mice. All the experiments were performed according to the ethical protocol authorised by the Italian Ministry of Health (n.1208/2020-PR).

Results

Intestinal Pgc1α is induced in liver steatosis and fibrosis

To investigate whether there is any correlation between MASLD and intestinal Pgc1α modulation, we analysed the mRNA expression of *Pgc1α* and its target genes in mice after the induction of liver steatosis and MASH. *Pgc1α* levels were significantly increased in the intestines of mice fed a Western diet (WD) for either 8 weeks (Fig. 1A and B) or 16 weeks (Fig. 1C and D). Whereas in the chow diet-fed mice the expression of the coactivator is mainly localised at the tip of the villi, as previously described,[11] in WD-fed mice Pgc1α is more scattered along the crypt–villus axis (Fig. 1B and D). In humans, obesity is frequently associated with liver steatosis and related complications. As bariatric surgery (Roux-en-Y gastric bypass) in grossly obese individuals is a way to decrease hepatic steatosis, inflammation, and fibrosis,[2] we analysed the transcriptional profile of jejunal biopsies from obese patients before and 1 month after gastric bypass (GSE113819), observing a significant downregulation of *PPARGC1A* expression[15] (Fig. S1A and B). Therefore, we may infer a negative correlation between the level of Pgc1α expression and the extent of liver steatosis and fibrosis in both mice and humans.

Intestinal-specific Pgc1α ablation affects gene expression

To explore the role of intestinal Pgc1α in the development of liver disorders, we generated iPgc1α$^{-/-}$ mice by crossing Pgc1α$^{fl/fl}$ mice with mice expressing Cre recombinase under Villin promoter to drive a specific intestinal deletion of exons 3–4–5 of Pgc1α gene (Fig. 1E). The specific deletion was confirmed by reverse-transcription quantitative PCR (RT-qPCR) for *Pgc1α* in the different intestinal tracts (Fig. 1F). RT-qPCR revealed a lower ileal expression of Pgc1α target genes Mitochondrial transcription factor A (*Tfam*), Cytochrome C (*Cyt-C*), and ATP synthase F1 subunit beta (*Atp5b*) in iPgc1α$^{-/-}$ mice compared with Pgc1α$^{fl/fl}$ littermates (Fig. 1G). Moreover, the ablation of Pgc1α determined a reduction in the mitochondrial endogenous, uncoupled, and Cox respiratory capacities in freshly isolated intact enterocytes (Fig. 1H). No difference in the expression of *Pgc1α* and its target genes was detected in the liver, white adipose tissue (WAT), and quadriceps by RT-qPCR (Fig. 1I–K).

To mimic the onset of MASLD, 2-month-old iPgc1α$^{-/-}$ and Pgc1α$^{fl/fl}$ littermates were fed a WD for 2 months. A regular chow diet was used as a control. No major modifications were detected in the intestinal architecture of the two genotypes at the time of sacrifice (Fig. 2A). Although a decreased length of intestinal villi was detected in iPgc1α$^{-/-}$ mice fed a chow diet, this difference became inconsistent following WD feeding (Fig. 2B).

To identify molecular pathways regulated by Pgc1α in response to WD, microarray analysis was performed on the ileum cells harvested from iPgc1α$^{-/-}$ and Pgc1α$^{fl/fl}$ mice after 2

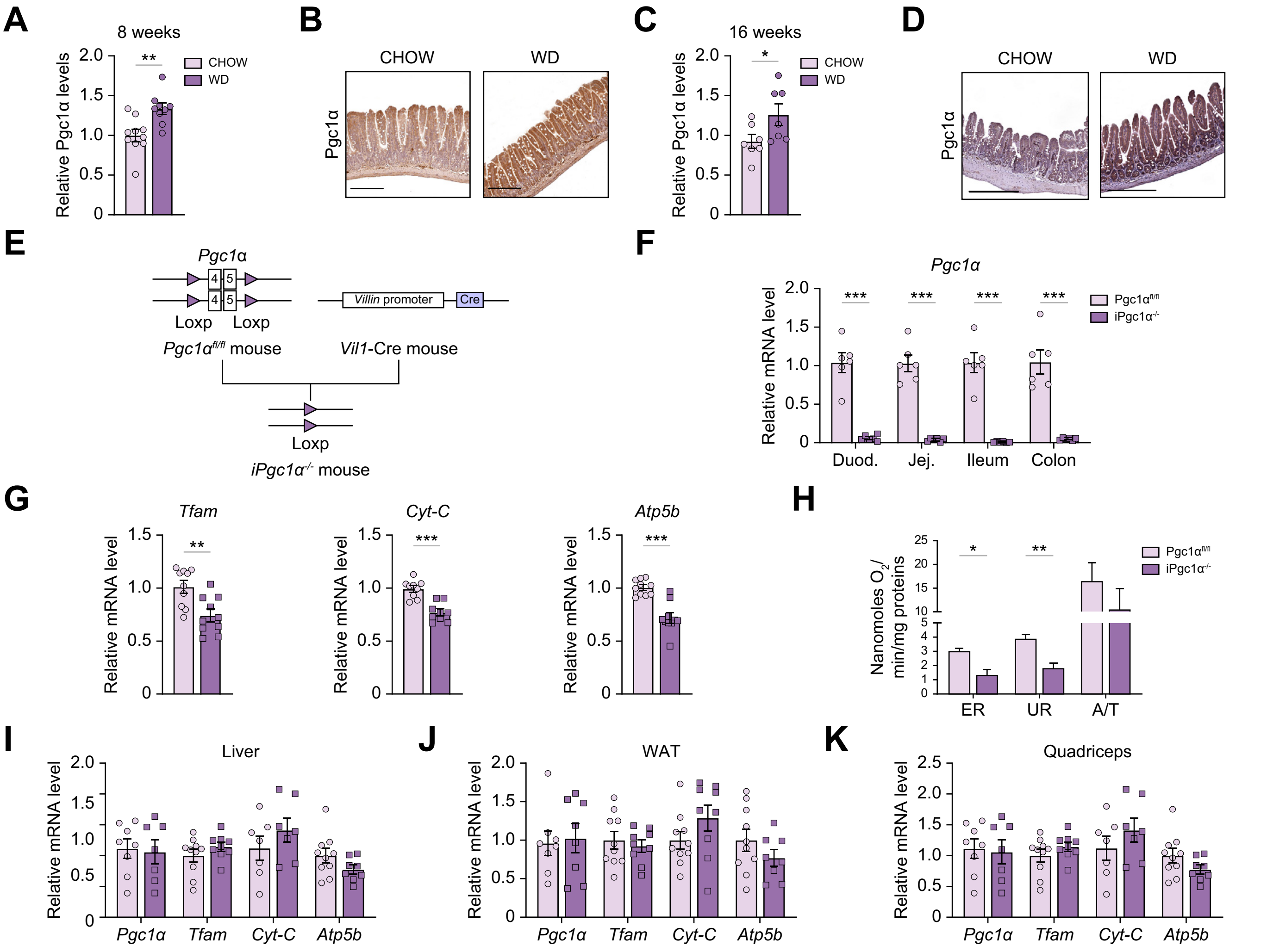

Fig. 1. Intestinal Pgc1α is induced in liver steatosis and fibrosis. Relative mRNA expression and immunohistochemistry of Pgc1α in the ileum of 2-month-old wild-type mice fed a chow diet or WD for (A, B) 8 weeks (scale bar, 200 μm) or (C, D) 16 weeks (scale bar, 300 μm). (E) Generation of iPgc1α$^{-/-}$ mice. (F) Relative Pgc1α expression in the different intestinal tracts. (G) Relative mRNA expression of Pgc1α target genes in the ileum. (H) Mitochondrial endogenous respiratory fluxes in enterocytes. Relative gene expression of Pgc1α and its target genes in (I) liver (J) WAT, and (K) quadriceps. All the experiments were performed on 4-month-old iPgc1α$^{-/-}$ and Pgc1α$^{fl/fl}$ mice (n = 6–10 animals/group). Data are expressed as mean ± SEM. Comparison between the two groups was performed using the Mann–Whitney *U* test (**p* <0.05; ***p* <0.01; ****p* <0.001). A/T, ascorbate/TMPD-dependent oxygen consumption; *Atp5b*, ATP synthase F1 subunit beta; *Cyt-C*, cytochrome C; DNP, 2,4 Dinitrophenol; ER, basal endogenous respiration; *Pgc1α*, peroxisome proliferator-activated receptor-gamma coactivator 1α; *Tfam*, mitochondrial transcription factor A; TMPD, N,N,N′,N′,-tetramethyl-phenylenediamine; UR, uncoupled respiration; WAT, white adipose tissue; WD, Western diet.

months of diet. The principal component analysis of the transcriptome revealed that diet is the principal component affecting gene expression, followed by the genotype (Fig. 2C). Among the differentially expressed genes in the comparison between the two diet treatments, up to 53% (993 genes) were common to both genotypes, whereas 25% (460 genes) and 22% (402 genes) were differentially expressed in Pgc1α$^{fl/fl}$ and iPgc1α$^{-/-}$ mice, respectively (Fig. 2D). Hierarchical clustering analysis of genes affected by WD in both genotypes identifies five clusters (Fig. 2E). Genes from clusters 2 and 5 were sensitive to the diet but not dependent on Pgc1α expression (Fig. 2F). Genes from cluster 4 display a difference between the two genotypes under only the chow diet regimen. The lack of Pgc1α altered the expression of genes in both diet conditions (clusters 1 and 3). Genes in cluster 1 are mainly induced in iPgc1α$^{-/-}$ animals and enriched for autophagy pathways. Intriguingly, genes in cluster 3 showed a decreased expression in iPgc1α$^{-/-}$ mice compared with Pgc1α$^{fl/fl}$ mice, with a marked downregulation as a result of WD consumption. Gene enrichment analysis revealed that genes of this cluster are mostly involved in the regulation of gene expression and translation (Fig. 2F).

As it has been described that an increased gut permeability may allow the passage of metabolites or inflammatory signals that favour the progression of liver diseases, we measured the mRNA levels of gap junction genes, known to tightly regulate paracellular permeability. The Claudin5 (*Cldn5*) gene displayed similar expression levels in all the groups analysed (Fig. S2A). Despite a reduced expression of Zonula Occuludens-1 (*Zo-1*) and

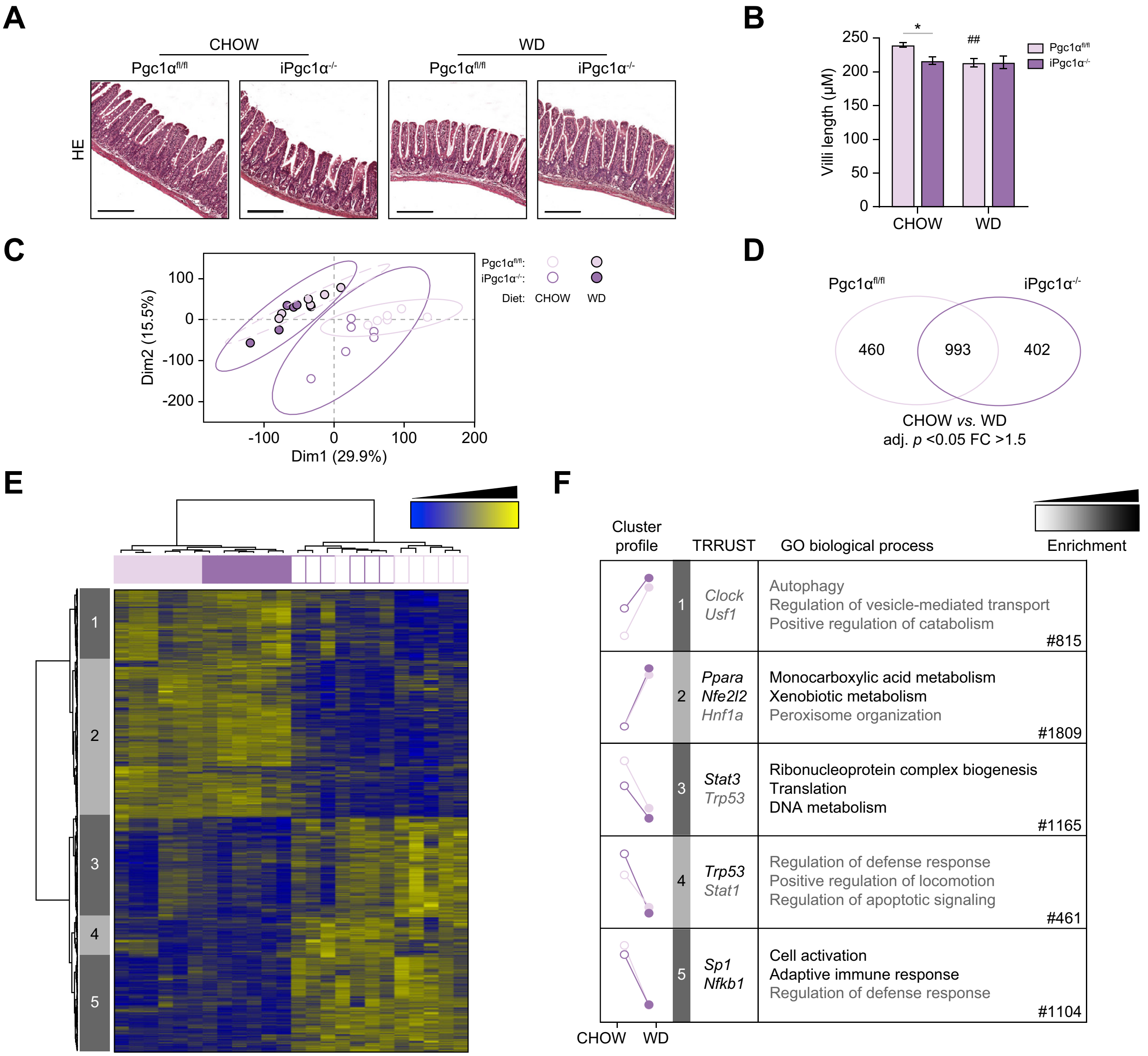

Fig. 2. Intestinal Pgc1α ablation does not impair intestinal architecture but affects gene expression. (A) Representative H&E of the ileum (scale bar, 200 μm). (B) Villi length assessed on 10 single complete full-size villi per sample. (C) PCA plots of the whole transcriptomic dataset in the intestine. Each dot represents an observation (animal) projected onto the first (horizontal axis) and second (vertical axis) PCA variables. (D) The number of genes differentially expressed between the two diets in Pgc1α$^{fl/fl}$ and iPgc1α$^{-/-}$ mice (adj. p <0.05, FC >1.5). (E) Heatmap showing data of microarray analysis on intestinal specimens. The hierarchical clustering identifies five different clusters. (F) Representation of the mean cluster profiles, the enrichment of transcription factors (TRRUST), the GO analysis, and the number of genes in each heatmap cluster. All the experiments were performed iPgc1α$^{-/-}$ and Pgc1α$^{fl/fl}$ littermates fed a chow diet or WD for 2 months (n = 6–10 animals/group). Data are expressed as mean ± SEM. Comparison between different groups was performed using two-way ANOVA followed by Sidak's multiple comparison tests; *genotype effect, #diet effect (* or #p <0.05; ** or ##p <0.01; *** or ###p <0.001). adj. p, adjusted p; FC, fold change; GO, Gene Ontology; PCA, principal component analysis; Pgc1α, peroxisome proliferator-activated receptor-gamma coactivator 1α; TRRUST, transcriptional regulatory relationships unravelled by sentence-based text-mining; WD, Western diet.

Occludin (*Ocln*) in iPgc1α$^{-/-}$ mice compared with controls in the chow diet, inconsistencies were detected between the two genotypes after WD administration (Fig. S2B). To further investigate whether the deletion of Pgc1α from the gut unbalances intestinal integrity, we measured plasma levels of FITC-conjugated dextran (Fig. S2C) and of lipopolysaccharide-binding protein (Fig. S2D), finding no differences in intestinal permeability between iPgc1α$^{-/-}$ and Pgc1α$^{fl/fl}$ mice in both diet conditions.

Overall, these results indicate that the ablation of Pgc1α from the intestinal epithelium drives major changes in the DNA-to-RNA process but does not impair intestinal permeability.

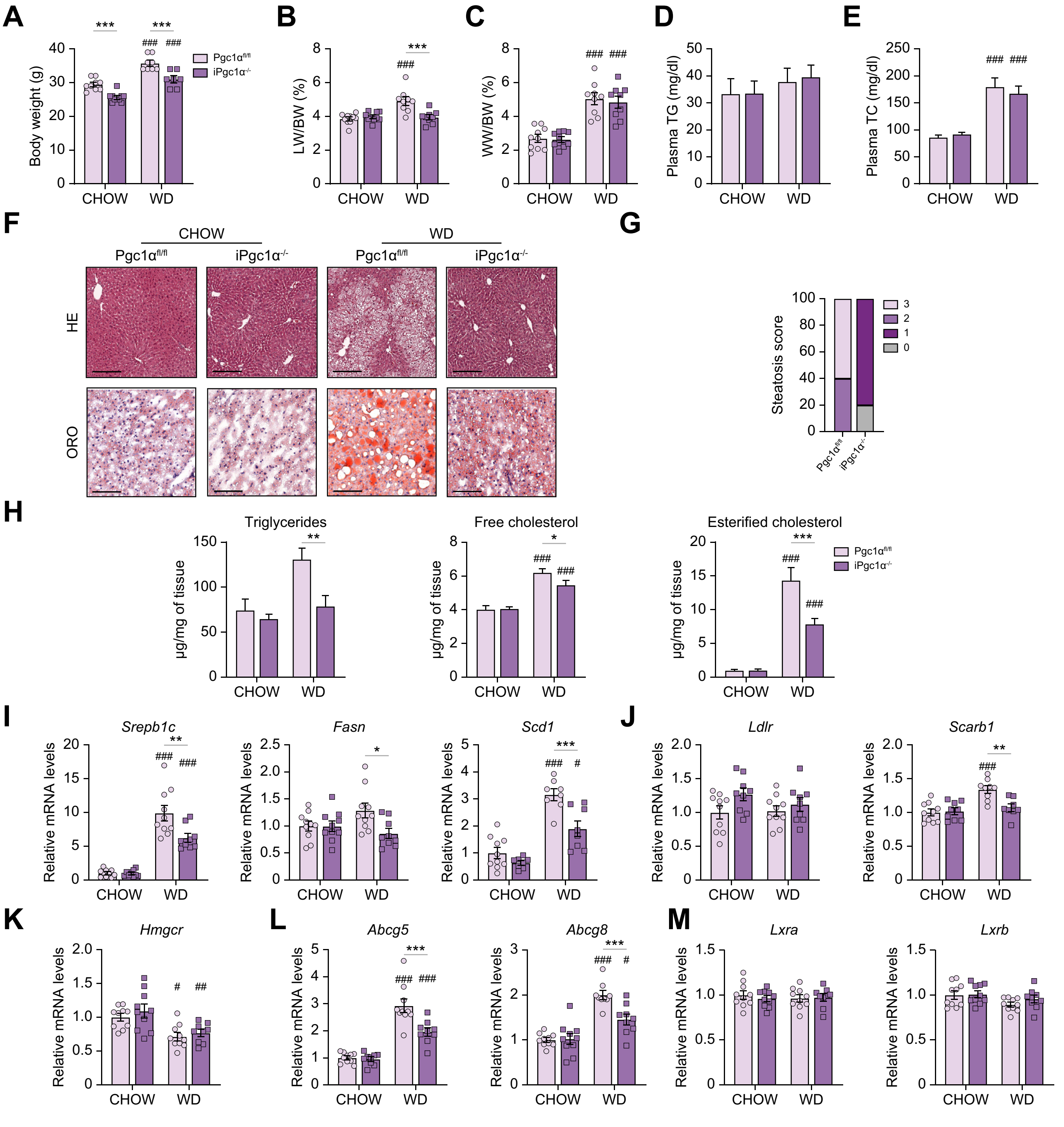

Fig. 3. Intestinal Pgc1α ablation protects from MASLD. (A) BW. (B) Relative LW and (C) WW expressed as a percentage to BW ratio. (D) Plasma TG and (E) TC. (F) Liver section stained with H&E and Oil Red O staining (scale bar, 100 μm). (G) Liver steatosis score based on parenchymal involvement by steatosis: 0, <5%; 1, 5–33%; 2, 33-66%; and 3, >66%. (H) Hepatic neutral lipids. Hepatic relative mRNA expression of genes involved in (I) *de novo* lipogenesis and (J–M) cholesterol export/uptake. All the experiments were performed on 4-month-old iPgc1α$^{-/-}$ and Pgc1α$^{fl/fl}$ littermates fed a chow diet or WD for 2 months (n = 6–10 animals/group). Data are expressed as mean ± SEM. Comparison between different groups was performed using two-way ANOVA followed by Sidak's multiple comparison tests; *genotype effect, #diet effect (* or #*p* <0.05; ** or ##*p* <0.01; *** or ###*p* <0.001). *Abcg5/8*, ATP-binding cassette G5/G8; BW, body weight; *Fasn*, fatty acid synthase; *Hmgcr*, 3-hydroxy-3-methylglutaryl-CoA reductase; *Ldlr*, LDL receptor; LW, liver weight; *Lxr*, liver X receptor; MASLD, metabolic dysfunction-associated steatotic liver disease; Pgc1α, peroxisome proliferator-activated receptor-gamma coactivator 1α; *Scarb1*, scavenger receptor class B type; *Scd1*, stearoyl-CoA desaturase 1; *Srebp1c*, sterol regulatory element-binding protein 1; TC, total cholesterol; TG, triglyceride; WD, Western diet; WW, white adipose tissue weight.

Intestinal-specific Pgc1α ablation protects against liver steatosis

After 2 months of WD, iPgc1α$^{-/-}$ mice displayed less body weight gain than Pgc1α$^{fl/fl}$ mice with comparable food consumption and a significantly lower liver-to-body weight ratio after WD feeding (Fig. 3A and B, and Fig. S3). The WAT-to-body weight ratio was affected by WD consumption, but not by genotype (Fig. 3C). Comparable levels of circulating triglycerides and cholesterol were detected among the two genotypes (Fig. 3D and E). H&E and Oil Red O staining showed a decreased lipid accumulation in iPgc1α$^{-/-}$ mice compared with controls (Fig. 3F), consistent with a lower steatosis score and a decreased accumulation of lipid specimens (triglycerides, and total and esterified cholesterol) within the liver (Fig. 3G and H). Given that reduced insulin sensitivity is frequently associated with MASLD, we assessed the glucose response in our mice. No remarkable changes in insulin sensitivity were detected between the two genotypes in both diet conditions or in the level of plasmatic incretin glucagon-like peptide 1 (Glp-1) (Fig. S4A–E).

To evaluate whether the lower steatosis observed in iPgc1α$^{-/-}$ mice was caused by changes in the synthesis of new fatty acids, we assessed the expression of Sterol regulatory element-binding protein 1 (*Srebp1c*), Fatty acid synthase (*Fasn*), and Stearoyl-CoA desaturase 1 (*Scd1*), observing a significantly decreased expression of the *de novo* lipogenesis genes in iPgc1α$^{-/-}$ mice compared with controls (Fig. 3I). No difference was detected in the expression genes responsible for *de novo* fatty acid synthesis in WAT (Fig. S5A), thus indicating the involvement of a specific hepatic mechanism. Once absorbed by the intestine, fatty acids are primarily stored in the WAT, from where they are released during lipolysis induced by fasting, driving a specific hepatic transcriptional response.[16] However, the expression levels of Adipose Triglyceride Lipase (*Atgl*), Hormone-Sensitive Lipase (*Hsl*), and Lipoprotein Lipase (*Lpl*) were not different between the two genotypes and are mainly modulated by diet consumption (Fig. S5B). Furthermore, to assess whether increased consumption of fatty acids by the muscle could be responsible for the observed phenotype, we measured the mRNA levels of fatty acid β-oxidation-related genes in the quadricep of our mice, finding no changes (Fig. S5C).

Next, we explored whether modifications of genes related to cholesterol metabolism were responsible for the decreased cholesterol accumulation observed. The expression of *Ldlr* and Scavenger receptor class B type (*Scarb1*), two major cholesterol importers, was mainly affected by genotype (Fig. 3J): whereas *Ldlr* levels were slightly induced in iPgc1α$^{-/-}$ mice in both diet conditions, the induction of *Scarb1* by the WD regimen was abolished in iPgc1α$^{-/-}$ mice as opposed to Pgc1α$^{fl/fl}$ mice. The mRNA levels of 3-hydroxy-3-methylglutaryl-CoA reductase (*Hmgcr*), codifying for the rate-limiting enzyme of cholesterol synthesis, were affected only by the diet, but not by the genotype (Fig. 3K). Finally, the expression of genes involved in cholesterol excretion from the hepatocytes, ATP-binding cassette G5 and G8 (*Abcg5/8*), was significantly reduced in iPgc1α$^{-/-}$ mice after WD (Fig. 3L). Altogether, this suggests that the intestinal-specific Pgc1α ablation protects against liver steatosis via modulation of liver X receptor (Lxr)-driven cholesterol and fatty acid metabolism. However, the mRNA level of *Lxrα* and *Lxrβ* did not reveal any alteration between the different groups (Fig. 3M), suggesting that the activity of Lxrs, rather than the expression, is involved in the observed phenotype. Indeed, as *Srepb1c*, *Fasn*, and *Abcg5/8* are all targets of Lxrs,[17,18] one could speculate that intestinal Pgc1α ablation decreased hepatic Lxr transcriptional activation via a reduction of their ligands, the cholesterol derivatives oxysterols.

Intestinal-specific Pgc1α ablation protects against steatohepatitis

To investigate whether the intestinal-specific Pgc1α ablation was still able to keep a protective phenotype in hepatic MASH, we sacrificed the mice after 4 months of diet. Consistently with steatosis data, iPgc1α$^{-/-}$ mice fed a WD displayed lower body weight and liver-to-body weight ratio, whereas the WAT-to-body weight ratio was influenced by only the diet (Fig. 4A–C). Moreover, the plasma alanine aminotransaminase level, a recognised marker of liver damage, was less elevated in iPgc1α$^{-/-}$ mice than in Pgc1α$^{fl/fl}$ mice in the WD condition (Fig. 4D).

Inflammation and fibrosis represent two hallmarks of MASH.[6] We first examined the distribution of liver macrophages with F4/80 immunostaining, a representative macrophage marker (Fig. 4E, upper panel). No considerable staining was detected in the liver of mice fed with a chow diet. In iPgc1α$^{-/-}$ mice, macrophages showed a scattered distribution in the liver section. In contrast, in Pgc1α$^{fl/fl}$ mice fed a WD, macrophages aggregated to surround hepatocytes with large lipid droplets, forming the hepatic crown-like structure (hCLS). Importantly, the hCLS represents a histological feature reflecting the extent of activation of Kupffer cells and liver fibrosis,[19] and their number was lower in the liver of iPgc1α$^{-/-}$ mice than in the liver of controls fed a WD (Fig. 4F), thus reflecting a diminished inflammatory and fibrotic process. In line with this, the expression of the macrophage surface marker *Cd68* was slightly decreased in iPgc1α$^{-/-}$ mice compared with Pgc1α$^{fl/fl}$ mice after WD feeding (Fig. 4H). Moreover, we observed a reduced mRNA level of M1 macrophage markers C-C motif chemokine ligand 2 (*Ccl2*) and tumor necrosis factor a (*Tnfa*) in iPgc1α$^{-/-}$ mice compared with control littermates (Fig. 4I). No differences were detected for interleukin1b (*Il1b*), whose expression is negatively affected by Lxr activity,[20] supporting once more our observations regarding the reduction of Lxr activation in iPgc1α$^{-/-}$ mice. The analysis of M2 macrophage markers (*Cd206* and Arginase 1, *Arg1*) revealed a trend to increase in iPgc1α$^{-/-}$ mice compared with controls, although not significant (Fig. 4J). A trend towards reduction in the expression of the hepatic stellate cell inducer transforming growth factor-beta (*Tgfb*) was detected in iPgc1α$^{-/-}$ mice compared with control littermates. To understand whether a different macrophage polarisation may be involved in our phenotype, we calculated the ratio between M1 and M2 macrophage markers (Fig. 4K). Interestingly, we observed that whereas Pgc1α$^{fl/fl}$ mice displayed an increased macrophage polarisation towards the M1 phenotype, usually associated with inflammation, iPgc1α$^{-/-}$ mice showed a significant reduction in the M1/M2 ratio, suggesting the occurrence of a more anti-inflammatory phenotype. Then, to dissect fibrosis, we examined collagen deposition in the liver of our mice. iPgc1α$^{-/-}$ mice fed a WD presented lower fibrosis than the control counterpart, as indicated by the Sirius Red staining (Fig. 4E and G) and the reduced mRNA level of genes involved in fibrosis (Fig. 4L). Overall, these data demonstrated that the intestinal-specific Pgc1α ablation confers protection also against liver fibrosis and inflammation.

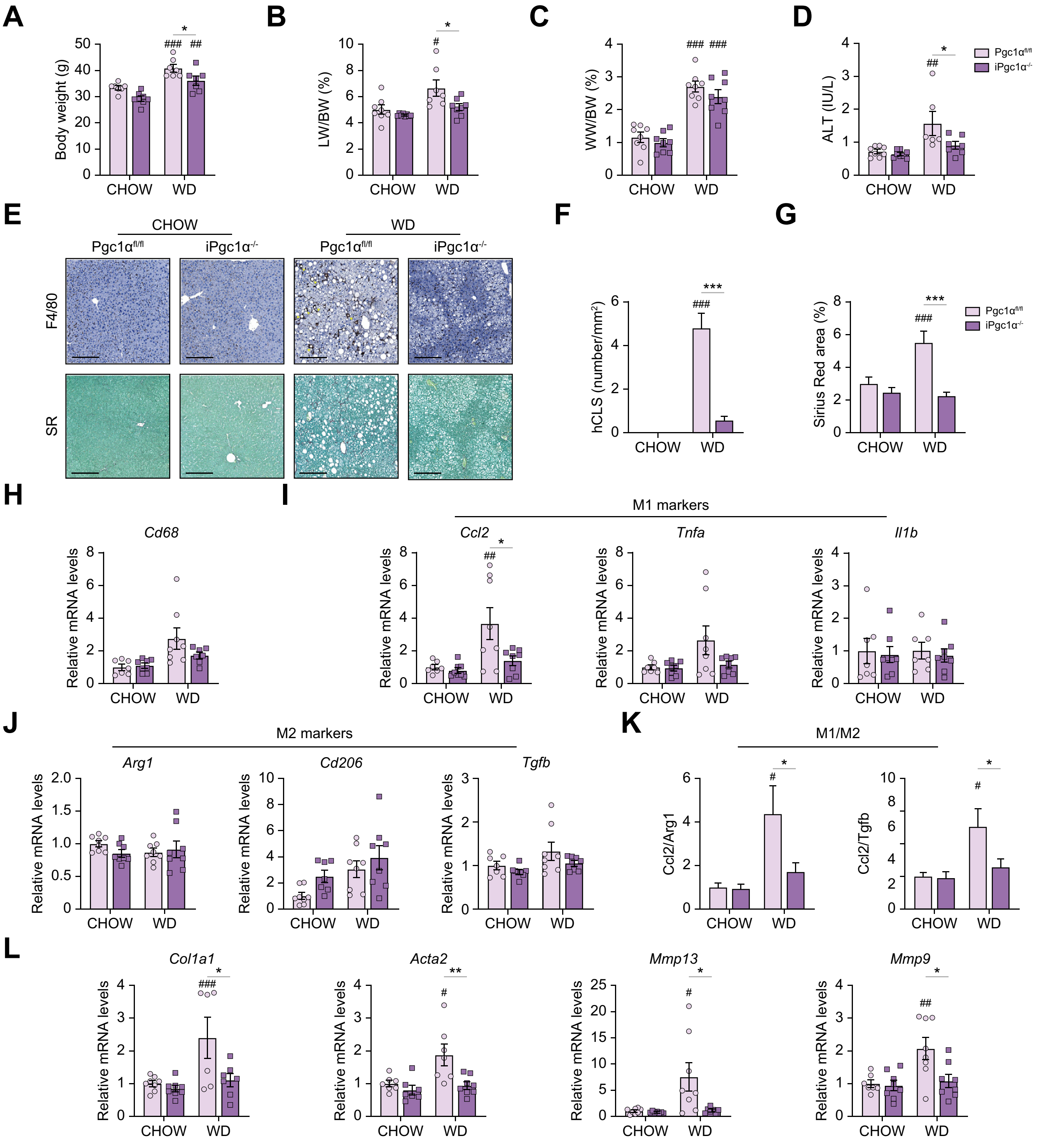

Fig. 4. Intestinal Pgc1α ablation protects from MASH. (A) BW. (B) Relative LW and (C) WW expressed as a percentage to BW ratio. (D) ALT level in plasma. (E) Liver section immunostained for F4/80 (upper panel) or stained with SR (lower panel) (scale bar, 100 μm). Yellow arrows indicate the hCLSs. (F) Quantification of hCLS as a number per mm^2. (G) Fibrosis quantified as the percent surface area occupied by SR-stained collagen. Hepatic relative mRNA expression of (H) Cd68 (I) M1 macrophage markers, and (J) M2 macrophage markers. (K) M1/M2 ratio. (L) Hepatic relative mRNA expression of genes involved in fibrosis. All the experiments were performed on 6-month-old iPgc1$\alpha^{-/-}$ and Pgc1$\alpha^{fl/fl}$ littermates fed a CD or WD for 4 months (n = 8 animals/group). Data are expressed as mean ± SEM. Comparison between different groups was performed using two-way ANOVA followed by Sidak's multiple comparison tests; *genotype effect, #diet effect (* or # *p* <0.05; ** or ## *p* <0.01; *** or ### *p* <0.001). *Acta2*, actin alpha 2; ALT, alanine aminotransferase; *Arg1*, Arginase 1; BW, body weight; *Ccl2*, C-C motif chemokine ligand 2; *Col1a1*, collagen type I alpha 1 chain; hCLS, hepatic crown-like structure; *I1b*, interleukin 1b; LW, liver weight; MASH, metabolic dysfunction-associated steatohepatitis; *Mmp 9/13*, matrix metalloproteinase 9/13; Pgc1α, peroxisome proliferator-activated receptor-gamma coactivator 1α; SR, Sirius Red; *Tgfb*, transforming growth factor-beta; *Tnfa*, tumor necrosis factor a; WD, Western diet; WW, white adipose tissue weight.

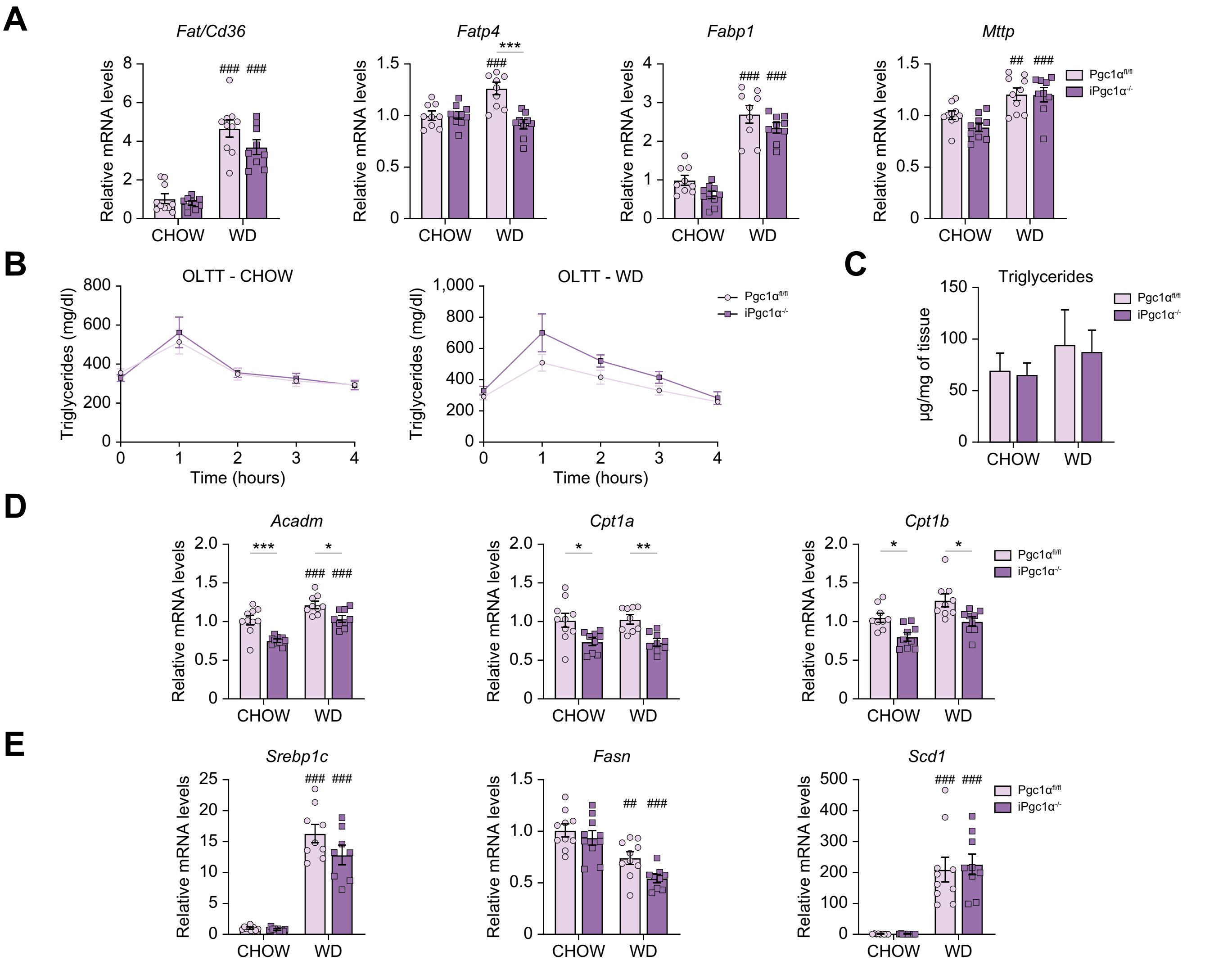

Fig. 5. Intestinal Pgc1α ablation does not impair fatty acid absorption. (A) Intestinal relative mRNA expression of genes involved in fatty acid absorption. (B) OLTT after a bolus of intralipid. (C) Intestinal triglyceride. (D) Intestinal relative mRNA expression of genes involved in fatty acid β-oxidation. (E) Intestinal relative mRNA expression of genes involved in *de novo* lipogenesis. All the experiments were performed on 4-month-old iPgc1α$^{-/-}$ and Pgc1α$^{fl/fl}$ littermates fed a chow diet or WD for 2 months (n = 10 animals/group). Data are expressed as mean ± SEM. Comparison between different groups was performed using two-way ANOVA followed by Sidak's multiple comparison tests; *genotype effect, #diet effect (* or # p <0.05; ** or ## p <0.01; *** or ### p <0.001). *Acadm*, acyl-CoA dehydrogenase medium chain; *Cpt1a/b*, carnitine palmitoyltransferase 1a/b; *Fabp1*, fatty acid-binding protein 1; *Fasn*, fatty acid synthase; *Fat/Cd36*, fatty acid translocase; *Fatp4*, fatty acid transporter 4; *Mttp*, microsomal triglyceride transfer protein; OLTT, oral lipid tolerance test; Pgc1α, peroxisome proliferator-activated receptor-gamma coactivator 1α; *Scd1*, stearoyl-CoA desaturase 1; *Srebp1c*, sterol regulatory element-binding protein 1; WD, Western diet.

Intestinal-specific Pgc1α ablation does not impair intestinal fatty acid absorption

The results obtained prompted us to investigate whether an impaired absorption of fatty acids driven by the absence of Pgc1α may explain the protective hepatic phenotype. To this end, we assessed the expression of the major fatty acid transporters in the intestine (Fig. 5A). WD feeding increases the mRNA levels of all the genes evaluated. Although no differences were observed for Fatty Acid Translocase (*Fat/Cd36*), Fatty Acid Binding Protein 1 (*Fabp1*), and Microsomal Triglyceride Transfer Protein (*Mttp*) between the two genotypes, the mRNA levels of Fatty Acid Transporter 4 (*Fatp4*) were significantly decreased in iPgc1α$^{-/-}$ mice fed a WD compared with controls. However, the oral lipid tolerance test did not display any difference in fatty acid absorption among the two genotypes in both diets (Fig. 5B). Moreover, the intestinal triglyceride level difference is comparable between the groups (Fig. 5C). A possible explanation may reside in the low expression of fatty acid β-oxidation genes driven by the absence of Pgc1α (Fig. 5D). Finally, we checked the level of genes involved in the *de novo* lipogenesis process (*Srebp1c, Fasn, and Scd1*), but the main differences observed were attributable more to the diet than to the genotype (Fig. 5E). Therefore, we may assume that Pgc1α does not affect intestinal fatty acid absorption.

Intestinal-specific Pgc1α ablation impairs cholesterol absorption

Given that we observed a reduction in the content of both cholesterol and esterified cholesterol in the liver of iPgc1α$^{-/-}$ mice fed a WD, we wonder whether Pgc1α may be implicated

in intestinal cholesterol trafficking. To this end, we measured the expression of genes regulating both cholesterol excretion and uptake in the intestine. Specifically, we analysed *Abcg5/8*, codifying for the sterol transporters that prevent the accumulation of dietary sterols, and ATP-binding cassette A1 (*Abca1*), which directly mediates cholesterol transport towards plasma HDL. The expression of all those genes was affected by WD regimen, but not by genotype (Fig. 6A). On the contrary, the level of *Scarb1* was diminished only in the enterocytes of iPgc1α$^{-/-}$ mice fed a chow diet compared with control mice, whereas no changes were detected during WD (Fig. 6B). As Scarb1 does not cause overall intestinal cholesterol absorption *in vivo*,[21] we analysed Niemann–Pick C1-like 1 (*Npc1l1*) mRNA level, which translates for a cholesterol transporter in the small intestine, finding a statistically significative reduction of this mRNA in iPgc1α$^{-/-}$ mice fed a WD compared with controls (Fig. 6C). No difference was detected in the intestinal cholesterol content, suggesting similar cholesterol retention between the two genotypes (Fig. 6D). To further validate this observation, we directly assessed the absorption of exogenous cholesterol by measuring serum fluorescence following oral delivery of a fluorescently labelled cholesterol mimetic. The ablation of Pgc1α correlates with a significantly decreased serum fluorescence after 8 h from administration (Fig. 6E), thus indicating an impaired cholesterol absorption in iPgc1α$^{-/-}$ mice.

Discussion

A critical aspect in the pathogenesis of hepatic steatosis is the development of inflammatory and fibrotic processes, which facilitate the progression towards severe liver diseases, such as cirrhosis and cancer.

The accumulation of harmful lipids is one of the factors that predispose the liver to develop steatohepatitis and its sequelae. It is worth noting that not all the classes of lipids drive lipotoxicity mechanisms, intimately associated with the establishment of chronic inflammation.[6] Excessive availability of cholesterol in the hepatocytes has deleterious effects, promoting cell damage and activating the fibrotic pathway.[4,5]

Although cholesterol can be endogenously synthesised, this process is disadvantageous for the cell, as it requires a considerable energetic expense. By contrast, large amounts of cholesterol can be easily accessed from the diet. In this view, the small intestine plays a unique role in cholesterol homoeostasis,[22] by regulating cholesterol absorption and excretion. Npc1l1 is the rate-limiting transporter of cholesterol in the small intestine, thus representing the pivotal regulator of cholesterol uptake from the gut lumen and – more in general – of systemic cholesterol homoeostasis. In humans, NPC1L1 is mainly expressed in the liver and the gut, but in murine models, its expression is limited to the gut.[23] Therefore, modifications in the activity of this transporter are directly attributable to the intestine.

Ezetimibe is an antihypercholesterolaemic drug that disrupts the structural cluster formed by NCP1L1 and cholesterol, thus leading to the inhibition of NPC1L1 functions.[24] Both genetic and pharmacological inactivation of Npc1l1 counteract the development of hepatic steatosis in mice fed high-fat diets, by decreasing the amount of hepatic cholesterol.[25,26] Moreover, also in humans, the ezetimibe treatment improves hepatic steatosis and inflammation in either obese individuals or non-obese ones with MASH,[27–29] although the results are not consistent with other clinical trials.[30]

In the current study, we found that an increased intestinal Pgc1α expression is associated with hepatic lipid accumulation,

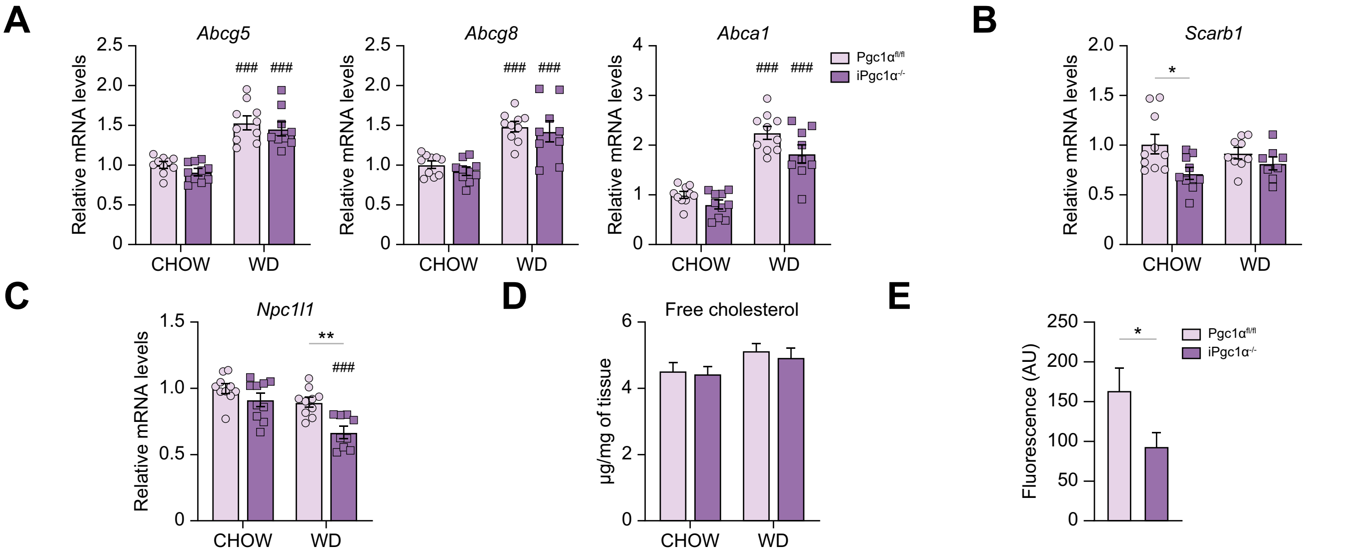

Fig. 6. Intestinal Pgc1α ablation impairs cholesterol uptake. (A–C) Intestinal relative mRNA expression of genes involved in cholesterol transport. (D) Intestinal free cholesterol. (E) Exogenous absorption of TopFluor cholesterol assessed after 8 h from administration. All the experiments were performed on 4-month-old iPgc1α$^{-/-}$ and Pgc1α$^{fl/fl}$ littermates fed a chow diet or WD for 2 months (n = 6–10 animals/group). Data are expressed as mean ± SEM. Comparison between different groups was performed using two-way ANOVA followed by Sidak's multiple comparison test or using Mann–Whitney *U* test; *genotype effect, #diet effect (* or #*p* <0.05; ** or ##*p* <0.01; *** or ###*p* <0.001). *Abca1*, ATP-binding cassette A1; *Abcg5/8*, ATP-binding cassette G5/G8; *Npc1l1*, Niemann–Pick C1-like 1; Pgc1α, peroxisome proliferator-activated receptor-gamma coactivator 1α; *Scarb1*, scavenger receptor class B type; WD, Western diet.

inflammation, and fibrotic processes in both humans and mice, thus priming the progression of metabolic liver diseases. The generation of a mouse model in which Pgc1α is specifically deleted from the gut reveals that the ablation of the coactivator does not impair intestinal architecture or gut permeability. Although this may appear contradictory with a previous study in which the expression of Pgc1α homologue was necessary to regulate intestinal integrity in old flies,[12] it has to be considered that our work was carried out in mice not older than 4–6 months and that ageing may be a crucial aspect to keep in consideration.

When we challenged the mice with a WD, we observed a dramatic reduction of hepatic steatosis, as a result of the lower accumulation of cholesterol and triglycerides in the hepatocytes (Fig. 3). Consistently, prolonged exposure to the diet results in lower hepatic inflammation and fibrosis, two aspects of MASH. Importantly, these effects seem to be driven specifically by the gut, given that we did not detect any alteration in systemic lipid disposal, as *de novo* lipogenesis or lipolysis processes in the WAT or altered fatty acid oxidation in the muscle. Remarkably, whereas we did not find any difference in the intestinal uptake of fatty acids, we observed a significant reduction of *Npc1l1* in the small intestine of animals lacking Pgc1α fed a WD, which led to an impaired cholesterol absorption (Fig. 6). The expression of *Npc1l1* is regulated by different transcription factors and nuclear receptors, including the Peroxisome proliferator-activated receptor α (Pparα), the Sterol regulatory element-binding protein 2 (Srebp2), and the Hepatocyte Nuclear Factor 4α (Hnf4α). Using HepG2 immortalised cell line, it has been demonstrated that *Npc1l1* expression can be induced either by Pparα/Retinoid X receptor-α (Rxrα) dimer or by Srebp2/Hnf4α dimer.[31,32] It is well recognised that Pgc1α acts as a coactivator of both Pparα and Hnf4α.[33,34] And, indeed, Pgc1α boosts the activity of Pparα/Rxrα and Srebp2/Hnf4α, stimulating the activation of *Npc1l1* transcription.[32] Although we did not assay these specific interactions in our study, this mechanism appears the most reliable one. Indeed, in our intestinal cells, we detected a significantly decreased expression of Pparα-regulated genes involved in fatty acid oxidation (Fig. 5D). However, we did not observe a reduction of *Fabp1* gene expression, as recently described, thus suggesting that probably other cofactors are needed to regulate that pathway.[7]

Several pieces of evidence demonstrated that dietary cholesterol promotes the hepatic build-up not only of cholesterol but also of triglyceride.[35] Our findings on iPgc1α$^{-/-}$ mice are in line with these observations. Indeed, the decreased cholesterol uptake from the gut results in a significant reduction of triglyceride accumulation in the liver as a result of a lower synthesis of new fatty acids. To note, ezetimibe treatment protects from MASLD by inhibiting Srebp1c, the master regulator of *de novo* lipogenesis.[36] It is possible that by inhibiting the intestinal cholesterol uptake, a downregulation of *Npc1l1* expression reduces cholesterol-dependent Lxr activation in the liver and the consequent induction of hepatic lipogenesis.[37–39] In our model, the expression of Lxrs target genes related to *de novo* lipogenesis as well as cholesterol secretion in the liver is impaired, thus corroborating the idea that the lack of Pgc1α in the gut disrupts the intestinal *Npc1l1* expression and reduces intestinal cholesterol absorption and subsequent hepatic lipid content via downregulation of Lxr transcriptome.

Recently, various compounds that target Pgc1α have been developed, and the beneficial effects on different conditions have been effectively evaluated. Among them, the small inhibitor SR-18292 improved the metabolic outcome of diabetes type 2, a condition frequently associated with MASLD and its sequelae, by blocking the gluconeogenic pathway and, more in particular, displacing the interaction between Pgc1α and Hnf4α.[40] Although the effects of this drug on the intestine have not been tested yet, it would be interesting to see whether they can disrupt the interaction between Pgc1α and Hnf4α also in the enterocytes, thus leading to lower *Npc1l1* expression.

In humans and mice, intestinal Pgc1α induction may be another culprit that drives WD-mediated liver steatosis and fibrosis. In the present study, we showed that enterocytes' specific ablation of Pgc1α protects from hepatic steatosis and fibrosis driven by the WD via a reduction of intestinal cholesterol absorption, and subsequent decrease of cholesterol and *de novo* fatty acid accumulation in the liver.

Abbreviations

Abca1, ATP-binding cassette A1; Abcg5/8, ATP-binding cassette G5/G8; Acadm, acyl-CoA dehydrogenase medium chain; Acta 2, actin alpha 2; ALT, alanine trasnaminase; Arg1, arginase 1; Atgl, adipose triglyceride lipase; Atp5b, ATP synthase F1 subunit beta; BW, body weight; CASH, cholesterol-associated steatohepatitis; Ccl2, C-C motif chemokine ligand 2; Cldn5, claudin5; Col1a1, collagen type I alpha 1 chain; Cpt, carnitine palmitoyltransferase; Cyt-C, cytochrome C; Fabp1, fatty acid-binding protein 1; Fasn, fatty acid synthase; Fat/Cd36, fatty acid translocase; Fatp4, fatty acid transporter 4; Glp-1, glucagon-like peptide 1; Hmgcr, 3-hydroxy-3-methylglutaryl-CoA reductase; Hnf4α, hepatocyte nuclear factor 4α; Hsl, hormone-sensitive lipase; Il1b, interleukin 1b; ITT, insulin tolerance test; Ldlr, LDL receptor; Lpl, lipoprotein lipase; LW, liver weight; Lxr, liver X receptor; MASH, metabolic dysfunction-associated steatohepatitis; MASLD, metabolic dysfunction-associated steatotic liver disease; Mmp, matrix metalloproteinases; Mttp, microsomal triglyceride transfer protein; NAFLD, non-alcoholic fatty liver disease; NASH, non-alcoholic steatohepatits; Npc1l1, Niemann–Pick C1-Like 1; Ocln, occludin; oGTT, oral glucose tolerance test; OLTT, oral lipid tolerance test; Pcsk9, pro-protein convertase subtilisin/kexin type 9; Pgc1α, peroxisome proliferator-activated receptor-gamma coactivator 1α; Pparα, peroxisome proliferator-activated receptor α; RT-qPCR, reverse-transcription quantitative PCR; Rxr, retinoid X receptor; Scarb1, scavenger receptor class B type; Scd1, stearoyl-CoA desaturase 1; Srebp1c, sterol regulatory element-binding protein 1; Tfam, mitochondrial transcription factor A; Tgfb, transforming growth factor-beta; Tnfa, tumor necrosis factor a; WAT, white adipose tissue; WD, Western diet; Zo-1, zonula occuludens-1.

Financial support

EP is funded by PON-AIM1853334, Attività 2-Linea 1. HG and NL are funded by Grant IMAGINE (ANR-20-CE14) from ANR. AM is funded by MIUR-PRIN n.2017J3E2W2; MIUR-PON 'R&I' 2014-2020 n. ARS01_01220; AIRC IG 2019 Id. 23239; HDHL-INTIMIC FATMAL-MIUR; and CN00000041, CUP H93C22000430007, Project title 'National Center for Gene Therapy and Drugs based on RNA Technology'. The project is funded under the National Recovery and Resilience Plan (NRRP), Mission 4, Component 2 Investment 1.4 – Call for tender no. 3138 of 16/12/2021 of the Italian Ministry of University and Research funded by the European Union – NextGenerationEU.

Conflicts of interest

All authors declare no conflict of interest.

Please refer to the accompanying ICMJE disclosure forms for further details.

Authors' contributions

Conceptualisation: EP, GV, AM. Data curation and formal analysis: EP, AP, YL. Investigation: EP, MA, MLM, MC, JB. Resources: HG, NL, AM. Visualisation: EP. Writing – original draft: EP, AM. Supervision: AM.

Data availability statement

The authors confirm that the data supporting the findings of this study are available within the article and/or its Supplementary materials and methods. Any additional data are available from the corresponding authors upon reasonable request. The data reported in this work have been uploaded to the Gene Expression Omnibus (GEO) database under accession number GSE227610.

Acknowledgements

A special thanks to Dr Roberta Le Donne for her invaluable help in administrative things. We are also particularly grateful to Dr Adina Pinna, Dr Ruggiero Di Paola, and Dr Federica Bevilacqua for the help provided in gene expression and histology experiments. We thank Claire Naylies for her contribution to microarray fingerprints acquisition and microarray data analysis carried out at GeT-TRiX, GeT Facility, Genopole Toulouse Midi-Pyrénées.

Supplementary data

Supplementary data to this article can be found online at https://doi.org/10.1016/j.jhepr.2023.100853.

References

Author names in bold designate shared co-first authorship

[1] **Younossi Z**, **Anstee QM**, **Marietti M**, **Hardy T**, **Henry L**, **Eslam M**, et al. Global burden of MASLD and MASH: trends, predictions, risk factors and prevention. Nat Rev Gastroenterol Hepatol 2018;15:11–20.
[2] Hafeez S, Ahmed MH. Bariatric surgery as potential treatment for nonalcoholic fatty liver disease: a future treatment by choice or by chance? J Obes 2013;2013:839275.
[3] Horn CL, Morales AL, Savard C, Farrell GC, Ioannou GN. Role of cholesterol-associated steatohepatitis in the development of MASH. Hepatol Commun 2022;6:12–35.
[4] Ioannou GN, Lee SP, Linsley PS, Gersuk V, Yeh MM, Chen YY, et al. *Pcsk9* deletion promotes murine nonalcoholic steatohepatitis and hepatic carcinogenesis: role of cholesterol. Hepatol Commun 2022;6:780–794.
[5] Wang X, Cai B, Yang X, Sonubi OO, Zheng Z, Ramakrishnan R, et al. Cholesterol stabilizes TAZ in hepatocytes to promote experimental non-alcoholic steatohepatitis. Cell Metab 2020;31:969–986.e967.
[6] Marra F, Svegliati-Baroni G. Lipotoxicity and the gut–liver axis in MASH pathogenesis. J Hepatol 2018;68:280–295.
[7] **Yan T**, **Luo Y**, Yan N, Hamada K, Zhao N, Xia Y, et al. Intestinal peroxisome proliferator-activated receptor α-fatty acid-binding protein 1 axis modulates nonalcoholic steatohepatitis. Hepatology 2023;77:239–255.
[8] Ducheix S, Piccinin E, Peres C, Garcia-Irigoyen O, Bertrand-Michel J, Fouache A, et al. Reduction in gut-derived MUFAs via intestinal stearoyl-CoA desaturase 1 deletion drives susceptibility to MASLD and hepatocarcinoma. Hepatol Commun 2022;6:2937–2949.
[9] **Piccinin E**, **Villani G**, **Moschetta A**. Metabolic aspects in MASLD, MASH and hepatocellular carcinoma: the role of PGC1 coactivators. Nat Rev Gastroenterol Hepatol 2019;16:160–174.
[10] D'Errico I, Lo Sasso G, Salvatore L, Murzilli S, Martelli N, Cristofaro M, et al. Bax is necessary for PGC1α pro-apoptotic effect in colorectal cancer cells. Cell Cycle 2011;10:2937–2945.
[11] D'Errico I, Salvatore L, Murzilli S, Lo Sasso G, Latorre D, Martelli N, et al. Peroxisome proliferator-activated receptor-γ coactivator 1-α (PGC1α) is a metabolic regulator of intestinal epithelial cell fate. Proc Natl Acad Sci U S A 2011;108:6603–6608.
[12] **Rera M**, **Bahadorani S**, **Cho J**, Koehler CL, Ulgherait M, Hur JH, et al. Modulation of longevity and tissue homeostasis by the *Drosophila* PGC-1 homolog. Cell Metab 2011;14:623–634.
[13] **Cunningham KE**, **Vincent G**, Sodhi CP, Novak EA, Ranganathan S, Egan CE, et al. Peroxisome proliferator-activated receptor-γ coactivator 1-α (PGC1α) protects against experimental murine colitis. J Biol Chem 2016;291:10184–10200.
[14] Pardo R, Enguix N, Lasheras J, Feliu JE, Kralli A, Villena JA. Rosiglitazone-induced mitochondrial biogenesis in white adipose tissue is independent of peroxisome proliferator-activated receptor γ coactivator-1α. PLoS One 2011;6:e26989.
[15] Ben-Zvi D, Meoli L, Abidi WM, Nestoridi E, Panciotti C, Castillo E, et al. Time-dependent molecular responses differ between gastric bypass and dieting but are conserved across species. Cell Metab 2018;28:310–323.e316.
[16] Fougerat A, Schoiswohl G, Polizzi A, Regnier M, Wagner C, Smati S, et al. ATGL-dependent white adipose tissue lipolysis controls hepatocyte PPARα activity. Cell Rep 2022;39:110910.
[17] Repa JJ, Berge KE, Pomajzl C, Richardson JA, Hobbs H, Mangelsdorf DJ. Regulation of ATP-binding cassette sterol transporters ABCG5 and ABCG8 by the liver X receptors α and β. J Biol Chem 2002;277:18793–18800.
[18] **Repa JJ**, **Liang G**, Ou J, Bashmakov Y, Lobaccaro JM, Shimomura I, et al. Regulation of mouse sterol regulatory element-binding protein-1c gene (SREBP-1c) by oxysterol receptors, LXRα and LXRβ. Genes Dev 2000;14:2819–2830.
[19] Ioannou GN, Haigh WG, Thorning D, Savard C. Hepatic cholesterol crystals and crown-like structures distinguish MASH from simple steatosis. J Lipid Res 2013;54:1326–1334.
[20] Thomas DG, Doran AC, Fotakis P, Westerterp M, Antonson P, Jiang H, et al. LXR suppresses inflammatory gene expression and neutrophil migration through cis-repression and cholesterol efflux. Cell Rep 2018;25:3774–3785.e3774.
[21] Altmann SW, Davis Jr HR, Yao X, Laverty M, Compton DS, Zhu LJ, et al. The identification of intestinal scavenger receptor class B, type I (SR-BI) by expression cloning and its role in cholesterol absorption. Biochim Biophys Acta 2002;1580:77–93.
[22] Lo Sasso G, Murzilli S, Salvatore L, D'Errico I, Petruzzelli M, Conca P, et al. Intestinal specific LXR activation stimulates reverse cholesterol transport and protects from atherosclerosis. Cell Metab 2010;12:187–193.
[23] Altmann SW, Davis Jr HR, Zhu LJ, Yao X, Hoos LM, Tetzloff G, et al. Niemann–Pick C1 like 1 protein is critical for intestinal cholesterol absorption. Science 2004;303:1201–1204.
[24] **Hu M**, **Yang F**, **Huang Y**, You X, Liu D, Sun S, et al. Structural insights into the mechanism of human NPC1L1-mediated cholesterol uptake. Sci Adv 2021;7:eabg3188.
[25] Davies JP, Scott C, Oishi K, Liapis A, Ioannou YA. Inactivation of NPC1L1 causes multiple lipid transport defects and protects against diet-induced hypercholesterolemia. J Biol Chem 2005;280:12710–12720.
[26] Nozaki Y, Fujita K, Yoneda M, Wada K, Shinohara Y, Takahashi H, et al. Long-term combination therapy of ezetimibe and acarbose for non-alcoholic fatty liver disease. J Hepatol 2009;51:548–556.
[27] Chan DC, Watts GF, Gan SK, Ooi EM, Barrett PH. Effect of ezetimibe on hepatic fat, inflammatory markers, and apolipoprotein B-100 kinetics in insulin-resistant obese subjects on a weight loss diet. Diabetes Care 2010;33:1134–1139.
[28] Enjoji M, Machida K, Kohjima M, Kato M, Kotoh K, Matsunaga K, et al. NPC1L1 inhibitor ezetimibe is a reliable therapeutic agent for non-obese patients with nonalcoholic fatty liver disease. Lipids Health Dis 2010;9:29.
[29] **Yoneda M**, **Fujita K**, Nozaki Y, Endo H, Takahashi H, Hosono K, et al. Efficacy of ezetimibe for the treatment of non-alcoholic steatohepatitis: an open-label, pilot study. Hepatol Res 2010;40:566–573.
[30] Loomba R, Sirlin CB, Ang B, Bettencourt R, Jain R, Salotti J, et al. Ezetimibe for the treatment of nonalcoholic steatohepatitis: assessment by novel magnetic resonance imaging and magnetic resonance elastography in a randomized trial (MOZART trial). Hepatology 2015;61:1239–1250.
[31] **Iwayanagi Y**, **Takada T**, Suzuki H. HNF4α is a crucial modulator of the cholesterol-dependent regulation of NPC1L1. Pharm Res 2008;25:1134–1141.
[32] **Iwayanagi Y**, **Takada T**, Tomura F, Yamanashi Y, Terada T, Inui K, et al. Human NPC1L1 expression is positively regulated by PPARα. Pharm Res 2011;28:405–412.
[33] Rhee J, Ge H, Yang W, Fan M, Handschin C, Cooper M, et al. Partnership of PGC-1α and HNF4α in the regulation of lipoprotein metabolism. J Biol Chem 2006;281:14683–14690.
[34] Vega RB, Huss JM, Kelly DP. The coactivator PGC-1 cooperates with peroxisome proliferator-activated receptor α in transcriptional control of nuclear genes encoding mitochondrial fatty acid oxidation enzymes. Mol Cell Biol 2000;20:1868–1876.

[35] Jia L, Betters JL, Yu L. Niemann-pick C1-like 1 (NPC1L1) protein in intestinal and hepatic cholesterol transport. Annu Rev Physiol 2011;73:239–259.

[36] Wang X, Sugimoto K, Fujisawa T, Shindo N, Minato S, Kamada Y, et al. Novel effect of ezetimibe to inhibit the development of non-alcoholic fatty liver disease in Fatty Liver Shionogi mouse. Hepatol Res 2014;44:102–113.

[37] Janowski BA, Willy PJ, Devi TR, Falck JR, Mangelsdorf DJ. An oxysterol signalling pathway mediated by the nuclear receptor LXRα. Nature 1996;383:728–731.

[38] **Grefhorst A**, **Elzinga BM**, Voshol PJ, Plosch T, Kok T, Bloks VW, et al. Stimulation of lipogenesis by pharmacological activation of the liver X receptor leads to production of large, triglyceride-rich very low density lipoprotein particles. J Biol Chem 2002;277:34182–34190.

[39] Betters JL, Yu L. NPC1L1 and cholesterol transport. FEBS Lett 2010;584:2740–2747.

[40] Sharabi K, Lin H, Tavares CDJ, Dominy JE, Camporez JP, Perry RJ, et al. Selective chemical inhibition of PGC-1α gluconeogenic activity ameliorates type 2 diabetes. Cell 2017;169:148–160.e115.

Journal of Hepatology, Volume *5*

Supplemental information

Intestinal Pgc1α ablation protects from liver steatosis and fibrosis

Elena Piccinin, Maria Arconzo, Maria Laura Matrella, Marica Cariello, Arnaud Polizzi, Yannick Lippi, Justine Bertrand-Michel, Hervé Guillou, Nicolas Loiseau, Gaetano Villani, and Antonio Moschetta

Supplementary Material

Intestinal Pgc1α ablation protects from liver steatosis and fibrosis

Elena Piccinin [1,2], Maria Arconzo [2], Maria Laura Matrella [1], Marica Cariello [2], Arnaud Polizzi [3], Yannick Lippi [3], Justine Bertrand-Michel [4], Hervé Guillou [3], Nicolas Loiseau [3], Gaetano Villani [1], Antonio Moschetta[2,5]*

[1] Department of Translational Biomedicine and Neuroscience (DiBraiN), University of Bari “Aldo Moro”, Bari, Italy
[2] Department of Interdisciplinary Medicine, University of Bari “Aldo Moro”, Bari, Italy
[3] Toxalim (Research Center in Food Toxicology), INRAE, ENVT, INP-PURPAN, UMR 1331, UPS, Université de Toulouse, Toulouse, France
[4] MetaboHUB-MetaToul, National Infrastructure of Metabolomics and Fluxomics, Toulouse, France
[5] INBB, National Institute for Biostructures and Biosystems, Rome, Italy

Table of contents

Supplementary Methods 2
Supplementary Figure 1 8
Supplementary Figure 2 9
Supplementary Figure 3 10
Supplementary Figure 4 11
Supplementary Figure 5 12
Supplementary Table 1 13
Supplementary Table 2 13
Supplementary References 14
CTAT methods **Errore. Il segnalibro non è definito.**

Supplementary Methods

Intestinal Permeability Assay

In vivo intestinal permeability was assessed using fluorescein isothiocyanate (FITC)-conjugated dextran (Sigma-Aldrich, USA; molecular mass 3–5 kDa). Mice were gavaged with 0.6mg/g body weight of FITC. After 4h blood was collected via submandibular puncture, and serum was collected by centrifugation. Serum fluorescence intensely correlating with intestinal permeability was measured in serum with the Microplate fluorometer VICTORTM EnLiteTM (PerkinElmer, Italy). Serum FITC concentration was calculated for each sample.

Glucose tolerance and insulin tolerance tests

For glucose tolerance tests, mice were fasted overnight before receiving an oral gavage with glucose at 2g/kg body weight. Blood glucose levels were measured before glucose administration (t=0) and then at times 15, 30, 60, and 120 minutes. For insulin tolerance tests, mice were fasted for 6h prior intraperitoneally injected with insulin at 0.5U/kg body weight. Blood glucose levels were measured before insulin administration (t=0) and then at times 15, 30, 60, and 120 minutes.

Insulin and GLP-1 assays

Mice were fasted overnight before receiving an oral gavage with glucose at 2g/kg body weight. Blood was collected from the submandibular vein a time 0, 15, and 30 minutes using Microvette serum tubes (Starsted, Germany). Insulin was assessed using Mouse Insulin ELISA (Mercodia, Sweden), following the manufacturer's instructions. GLP-1 levels were measured with Multi Species GLP-1 Total ELISA (Merck, Germany) following the manufacturer's instructions.

Oral lipid tolerance test

Mice were fasted overnight before receiving an oral gavage of intralipid 20 (Sigma-Aldrich, USA) 10mL/kg body weight. Blood was then collected from the tail vein and centrifuged (1000×g, 10 min at 4°C) to obtain plasma before and at 60, 120, 180, and 240 minutes after the administration of the oil. Plasma triglyceride levels were immediately measured using a commercial kit (Sentinel Diagnostic, Italy).

Cholesterol transport assay

Intestinal uptake of cholesterol was measured using TopFluor-cholesterol (Avanti Polar Lipids, Alabaster, AL) in mice fed a chow diet. To mimic intestinal lipid processing, a lipid mixture of TopFluor-cholesterol (8 µg/gm body weight), phospholipids (Sigma-Aldrich, St. Louis, MO), and taurocholate (Sigma-Aldrich, St. Louis, MO) was obtained, generating a so-called a bile model [1]. To this mixture, methyl-β-cyclodextrin (Sigma-Aldrich, St. Louis, MO) was added since it is necessary to deplete already present plasma membrane cholesterol [2]. Blood was collected from the submandibular vein using Microvette serum tubes (Starsted, Germany) at times 0 and 8 hours. Serum fluorescence was read at Microplate fluorometer VICTORTM EnLiteTM (PerkinElmer, Italy). Samples with detectable hemolysis were excluded from the analysis.

Organs and blood sampling

At of sacrifice, tissues were removed, dissected, snap-frozen in liquid nitrogen, and stored at -80°C until use. For the ileum section, the isolation of single cells was performed by a modification of the Weiser method, as previously described [3, 4]. Briefly, the ileum was harvested, washed with a cold saline solution, and cut longitudinally. To remove the luminal content, the ileum was transferred into Hank's balanced salt solution with 0.5 mM DTT. Then, ileum was placed into 20 ml of Chelating Buffer (Na2PO4 5mM, NaCl 96mm, Na Citrate27mM, KH2PO4 8mM, KCl 1.5 mm, D-Sorbitol 55mM, Sucrose 44mM, DTT 0.5 mM), and incubated at 4˚C with constant stirring for 20 minutes and the supernatant was collected. After, the ileum was transferred into a 50ml tube with 15 ml of Chelating buffer, gently shaken by hand (30 inversions) and the supernatant was collected. This operation was repeated eight times. All collected supernatants were centrifuged at 1000g, washed with cold saline buffer, and then stored at -80°C. Blood was collected by cardiac puncture using heparin-coated syringes. Plasma was prepared by centrifugation (1000×g, 10 min at 4°C) and kept at -80°C until use.

Measurement of Endogenous Respiratory Fluxes in Intact Enterocytes

Respiration rates were measured polarographically with a Clark-type oxygen electrode in a water-jacketed chamber (Hansatech Instruments), magnetically stirred at 37 °C as previously described [3]. Briefly, pooled fractions of freshly isolated enterocytes were collected by centrifugation and then were transferred into the polarographic chamber at 1–2 mg of protein/mL in TD buffer [0.137 M NaCl, 5 mM KCl, 0.7 mM Na2HPO4, 25 mM Tris·HCl (pH 7.4)] air-equilibrated at 37 °C. The respiration rate by endogenous substrates was read directly (endogenous respiration) and after the addition of 60 µM 2,4-dinitrophenol

(uncoupled respiration). The in-situ cytochrome c oxidase activity (A-T) was measured as the KCN-sensitive oxygen consumption rate elicited by 10 mM sodium ascorbate + 0.4 mM N,N,N′,N′,-tetramethyl-phenylenediamine in the presence of antimycin A.

Gene Expression

For the liver, ileum single cells, and gastrocnemius, total RNA was extracted with Qiazol reagent (Qiagen, Germany). For inguinal white adipose tissue, total RNA was extracted using the RNeasy Lipid Tissue Mini Kit (Qiagen, Germany), following the instructions. 1-2 µg of total RNA were treated with DNase (Thermo Fisher Scientific, USA) and retrotranscribed to cDNA using the High Capacity cDNA Reverse Transcription Kit (Thermo Fisher Scientific, Massachusetts, USA) following the manufacturer’s instructions. qPCR assays were performed in 96-well plates using the Master Mix Power SYBR Green (Thermo Fisher Scientific, USA) via the QuantStudio5 machine (Thermo Fisher Scientific, USA), and the first analysis was performed using the QuantStudio Design & Analysis. Relative quantification was calculated via the ΔΔCT method, using Tbp (TATA-binding protein) as a reference gene. All the primers used in this study are listed in Supplementary Table 1.

Transcriptome profiles were obtained for 6 intestinal samples per group at the GeT-TRiX facility (GénoToul, Génopole Toulouse Midi-Pyrénées) using Sureprint G3 Mouse GE v2 microarrays (8 3 60K, design 074809, Agilent Technologies), according to the manufacturer’s instructions. For each sample, Cyanine-3 (Cy3) labelled cRNA was prepared from 200 ng of total RNA using the One-Color Quick Amp Labeling kit (Agilent Technologies), according to the manufacturer’s instructions, followed by Agencourt RNAClean XP (Agencourt Bioscience Corporation, Beverly, Massachusetts). Dye incorporation and cRNA yield were determined using a Dropsense 96 UV/VIS droplet reader (Trinean, Belgium). Next, 600 ng of Cy3-labeled cRNA were hybridized on the microarray slides, following the manufacturer’s instructions. Immediately after washing, slides were scanned on an Agilent G2505C Microarray Scanner using Agilent Scan Control A.8.5.1 software, and the fluorescence signal was extracted using Agilent Feature Extraction software v10.10.1.1 with default parameters. Microarray data and experimental details are available in NCBI’s Gene Expression Omnibus (GEO) database (accession numbers GSE227610).

Publicly available mRNA expression profiles were obtained by gene expression omnibus (GEO Accession Number: GSE113819, www.ncbi.nlm.nih.gov/geo/query/acc.cgi?acc=GSE113819). Specifically, since the search terms “NAFLD” OR “NASH” OR “liver steatosis” OR “steatohepatitis” AND “small intestine” AND “homo sapiens” do not retrieve any information, we used the search terms “obesity”

AND “small intestine” AND “homo sapiens”, recovering 7 different datasets. Datasets with specimens from other organisms, expression profiles by RT-qPCR or RNA sequencing, or the absence of a clear control group were excluded.

Microarray Analysis

Microarray data were analyzed using R and Bioconductor packages [5]. Raw data (median signal intensity) were filtered, log2 transformed, corrected for batch effects (microarray washing bath and labelling serials), and normalized using the qsmooth method [6]. A model was fitted using the limma lmFit function [7]. Pairwise comparisons between biological conditions were applied using specific contrasts. A correction for multiple testing was applied using the Benjamini-Hochberg procedure to control the false discovery rate (FDR). Probes with an FDR≤0.05 were considered to be differentially expressed between conditions. Hierarchical clustering was applied to the samples and the differentially expressed probes using 1-Pearson correlation coefficient as distance and Ward’s criterion for agglomeration. The clustering results are illustrated as a heatmap of expression signals. Gene ontology and transcription factor enrichment analysis were performed using Metascape [8].

Histology and Immunohistochemistry

Tissue specimens were fixed in 10% formalin for 12-24 hours, dehydrated, and paraffin-embedded. Ileum and liver sections (2 µm) were stained with hematoxylin-eosin staining (HE), according to the standard procedures. Villi length was calculated by evaluating complete, full-sized intestinal villi (n=10) not exhibiting bending or mechanical damage, for each sample. Sirius Red staining using Direct Red 80 and Fast Green FCF (Sigma-Aldrich, USA) was performed on liver sections to assess fibrosis. Immunohistochemistry analysis was performed in liver and ileum specimens (4 µm). Briefly, sections were subjected to antigen retrieval by boiling the slides in sodium citrate pH 6 for 15 minutes, permeabilized in phosphate-buffered saline with 0.25% Triton X-100 for 5 minutes, and then incubated for 10 minutes at room temperature in protein blocking solution (Dako, Denmark). Subsequently, sections were incubated with primary antibodies as indicated in Supplementary Table 2. Sections were washed in PBS for 15 minutes and incubated at room temperature with DAKO real EnVision detection system (Dako, Denmark), according to the manufacturer’s instruction. For negative controls, 1% nonimmune serum in PBS substituted the primary antibodies. Images were acquired and analyzed with Aperio Image Scope (Leica Biosystems, Germany). The percentage of stained area/total area was evaluated in 10 consecutive acquired images. Values from all consecutive images for each sample were

averaged and displayed as mean±SEM. Steatosis score was assigned based on the percentage of hepatic parenchyma containing fat: 0 - <5%; 1 – 5-33%; 2 – 33-66%; 3 - >66% [9].
Specimens from livers were embedded in OCT (Sakura), frozen under nitrogen vapours, and stored at –80°C. Liver cryosections (4 µm) were stained with Oil Red O Stain Kit (ab150678, Abcam, UK) following the manufacturer's indications.

Lipidomic Assay

Fatty acids were extracted from frozen tissues or plasma using a modified Bligh and Dyer extraction method. Samples were lysed in a water EDTA (5 Mm)/methanol mix (1:2 vol/vol). Methanol and dichloromethane were added to reach the following ratios of MeOH/water/CH2Cl2: 2.5/2.0/2.5. Glyceryltrinonadecanoate was added as an internal standard. The dried lipid extract was transmethylated with 1 ml of BF3 in methanol (1:20, vol/vol) for 60 min at 100°C, evaporated to dryness, and the fatty acid methyl esters (FAMEs) were extracted with hexane/water (3:1). The organic phase was evaporated to dryness and dissolved in 50 µl ethyl acetate. FAMEs were analyzed by gas-liquid chromatography on a 5890 Hewlett-Packard system using a Famewax fused-silica capillary column (30 m, 0.32 mm i.d., 0.25-mm film thickness; Restek). The oven temperature was programmed from 110ºC to 220°C at a rate of 2°C/min, and the carrier gas was hydrogen (0.5 bar). The injector and the detector were at 225ºC and 245°C, respectively. Neutral lipids were extracted from plasma, liver or intestine frozen tissues using a Bligh and Dyer extraction method: samples were homogenized in methanol/5 mM EGTA (2:1, v/v), and lipids (corresponding to an equivalent of 2mg tissue) extracted according to the Bligh–Dyer method63, with chloroform/methanol/water (2.5:2.5:2 v/v/v), in the presence of the following internal standards: glyceryl trinonadecanoate, stigmasterol, and cholesteryl heptadecanoate (Sigma-Aldrich). Triglycerides, free cholesterol, and cholesterol esters were analyzed by gas-liquid chromatography on a Focus Termo Electron system equipped with a Zebron- 1 Phenomenex fused-silica capillary column (5 m, 0.25 mm i.d., 0.25 mm film thickness). The oven temperature was programmed to increase from 200 to 350 °C at 5 °C/min, and the carrier gas was hydrogen (0.5 bar). The injector and detector temperatures were 315 °C and 345 °C, respectively.

Statistical Analysis

All the results are expressed as mean ± SEM. Statistical analyses were performed with GraphPad Prism software analysis (v9.0, GraphPad Software, USA). Outliers were

calculated with ROUT or Grubbs test. To compare two groups Mann Whitney U test was used, while for four groups 2-Way ANOVA followed by Tukey's post-hoc test. A p-value <0.05 was considered significant. Paired T-Test was used to analyze paired data. Significant genotype effect was indicated by * (*p < 0.05, **p < 0.01, ***p < 0.001). Significant diet effect was indicated by # ($^{\#}p < 0.05$, $^{\#\#}p < 0.01$, $^{\#\#\#}p < 0.001$).

Supplementary Figure 1

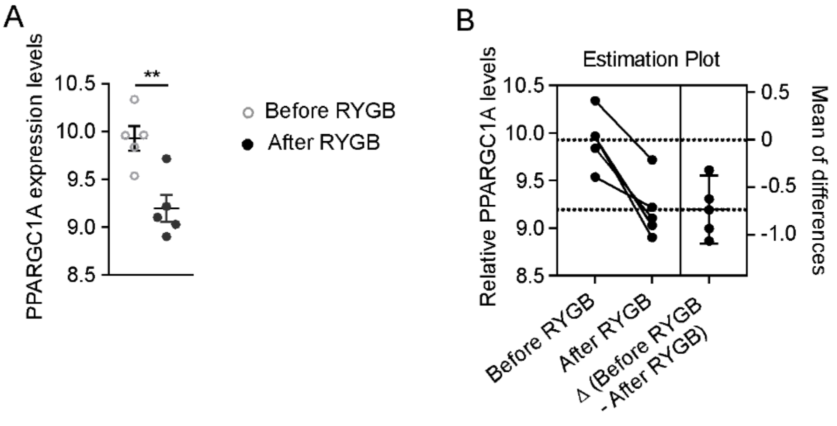

Supplementary Figure 1. PPARGC1A expression is increased in obese individuals. (A) PPARGC1A expression levels in small intestine harvested from obese patients before and after RYGB bypass (GSE113819) and (B) estimation plot of the PPARGC1A expression: on the left, scatter plot showing individual points; on the right, the effect size (difference between means). Data are expressed as mean±SEM. Comparison between distinct groups was performed using Paired T-test (**p<0.01).

Supplementary Figure 2

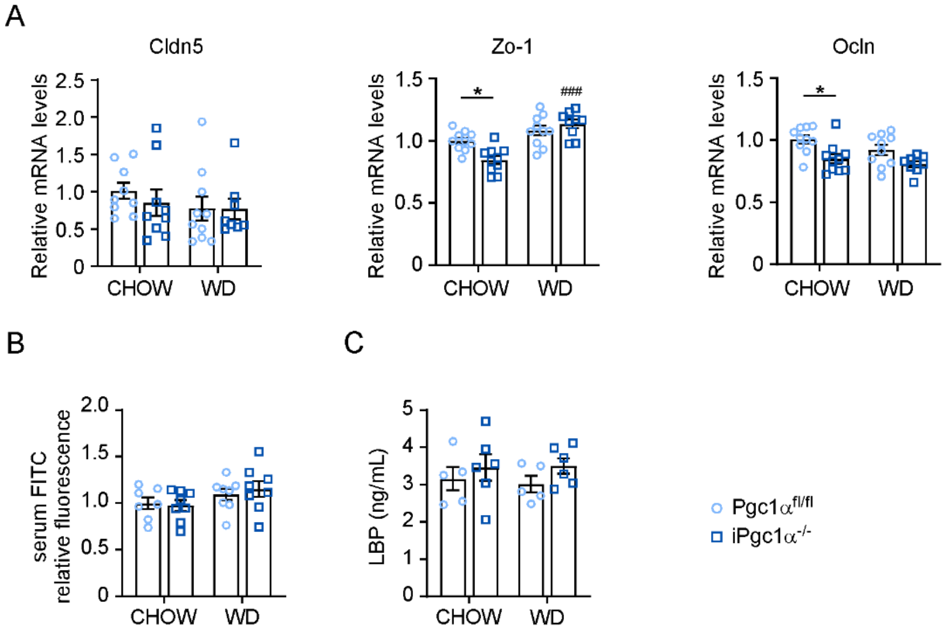

Supplementary Figure 2. Pgc1α ablation does not impair intestinal permeability. (A) Relative mRNA expression of tight junction proteins, Claudin 2 (Cldn5), Zona Occludens 1 (Zo-1), and Occludin (Ocln) measured in the ileum. (B) *In vivo* intestinal permeability measured by FITC assay. (E) Lipopolysaccharide binding protein measured in plasma. All the experiments were performed on 2 months old iPgc1α$^{-/-}$ and Pgc1α$^{fl/fl}$ littermates fed with chow or western diet (WD) for 2 months. (n=6-10 animals/group). Data are expressed as mean±SEM. Comparison between distinct groups was performed using Two-way ANOVA followed by Sidak's multiple comparison test; *genotype effect $^{\#}$diet effect (* or $^{\#}$p<0.05; ** or $^{\#\#}$p<0.01; *** or $^{\#\#\#}$p<0.001).

Supplementary Figure 3

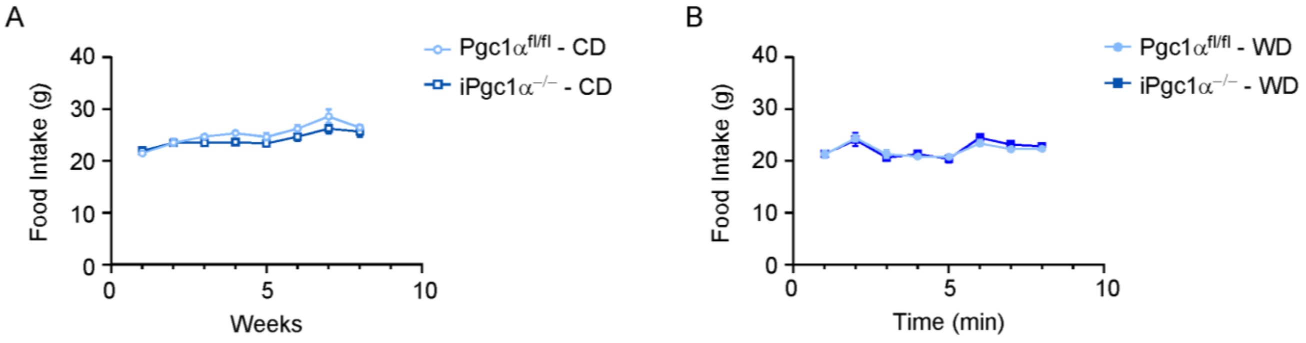

Supplementary Figure 3. Intestinal Pgc1α ablation does not alter food intake. Food intake of iPgc1α$^{-/-}$ mice and their Pgc1α$^{fl/fl}$ littermates fed (A) chow diet and (B) western diet (WD) for 2 months. All the experiments were performed iPgc1α$^{-/-}$ and littermates' control Pgc1α$^{fl/fl}$ (n=10 animals/group). Data are expressed as mean±SEM. Comparison between two groups was performed using Multiple Mann Whitney Test.

Supplementary Figure 4

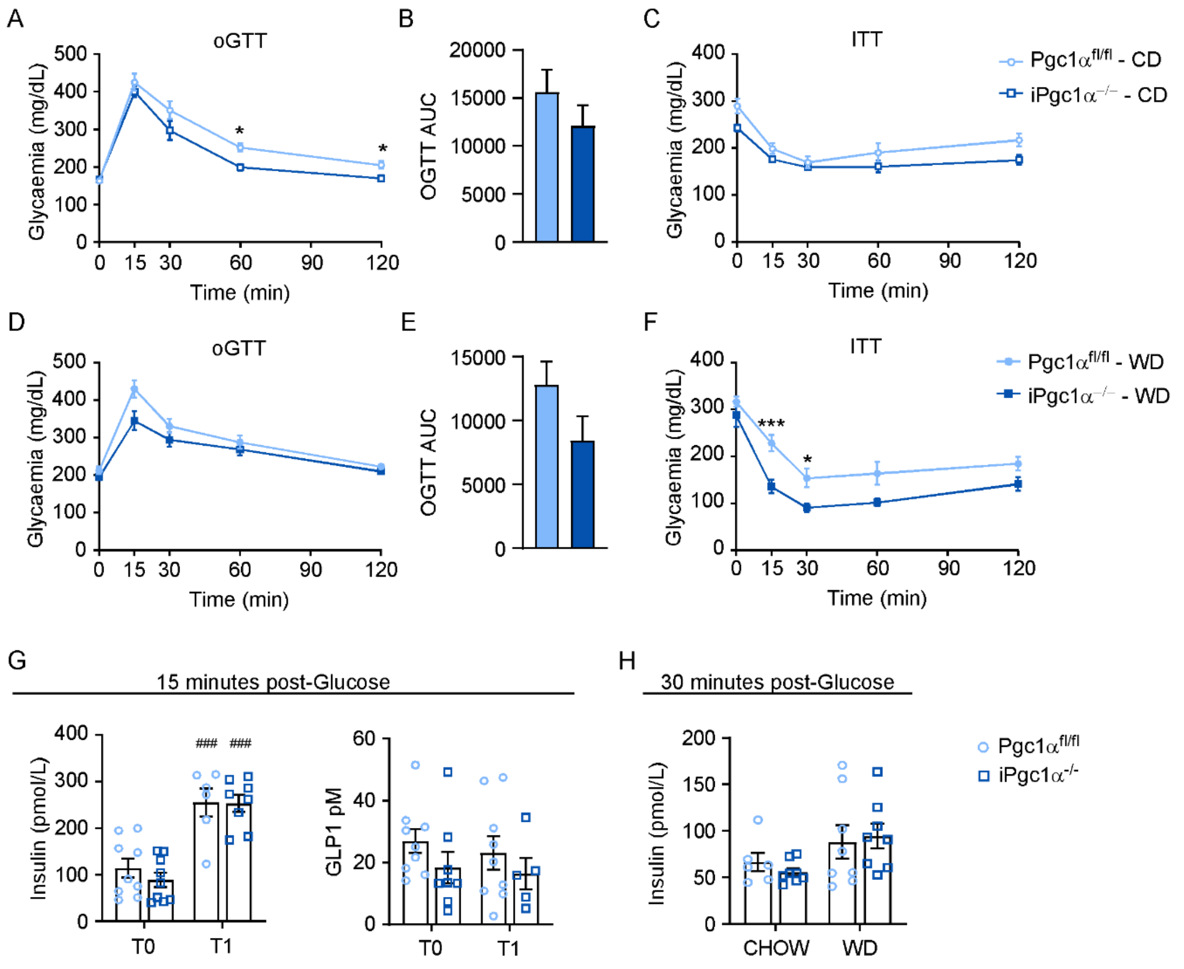

Supplementary Figure 4. Intestinal Pgc1α ablation does not alter glucose tolerance. (A) Oral glucose tolerance test, (B) relative Area Under the Curve (AUC), and (C) insulin tolerance test performed in fasted chow diet fed animals. (D) Oral glucose tolerance test, (E) relative AUC, and (F) insulin tolerance test performed in fasted western diet fed animals. (G) Serum insulin and GLP1 levels at basal level and 15 minutes after an intragastric bolus of glucose in fasted chow diet fed animals. (H) Serum insulin levels 30 minutes after an intragastric bolus of glucose in fasted chow diet and western fed animals. All the experiments were performed on 2 months old iPgc1α$^{-/-}$ mice and their Pgc1α$^{fl/fl}$ littermates were fed with chow or western diet (WD) (n=10 animals/group). Data are expressed as mean±SEM. Comparison between distinct groups was performed using Two-way ANOVA followed by Sidak's multiple comparison test; *genotype effect $^{\#}$diet effect (* or $^{\#}$p<0.05; *** or $^{\#\#\#}$p<0.001).

Supplementary Figure 5

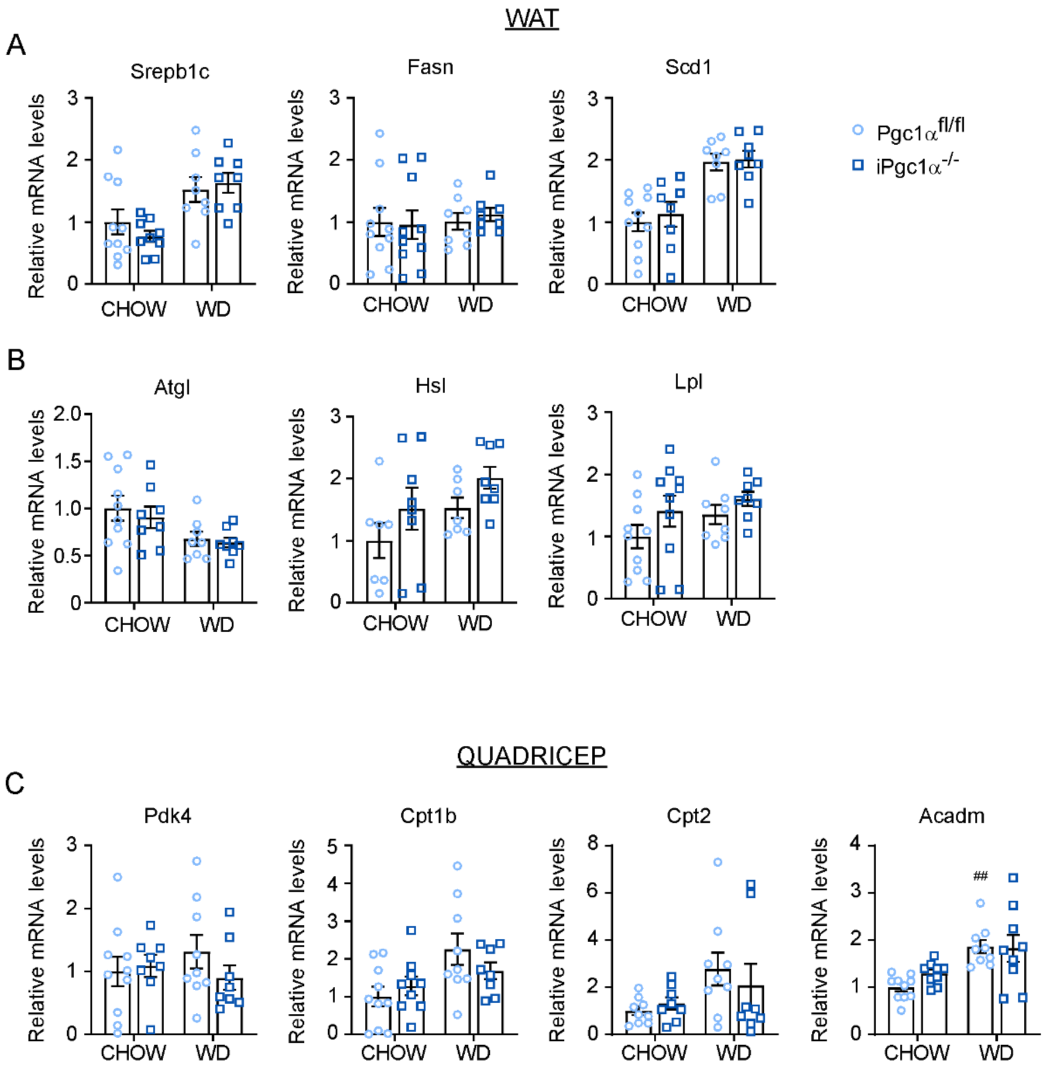

Supplementary Figure 5. Intestinal Pgc1α ablation does not modulate white adipose tissue and muscle gene expression. (A) Relative mRNA expression of genes involved in de novo lipogenesis in the WAT. (B) Relative mRNA expression of genes involved in lipolysis in the WAT. (C) Relative mRNA expression of genes involved in fatty acids β-oxidation in the quadriceps. All the experiments were performed on 2 months old iPgc1α$^{-/-}$ mice and their Pgc1α$^{fl/fl}$ littermates were fed with chow or western diet (WD) (n=10 animals/group). Data are expressed as mean±SEM. Comparison between distinct groups was performed using Two-way ANOVA followed by Sidak's multiple comparison test.

Supplementary Table 1
List of primers used in the study.

Gene	Sequence Primer Forward	Sequence Primer Reverse
Abcg5	TCAATGAGTTTTACGGCCTGAA	GCACATCGGGTGATTTAGCA
Abcg8	AATGTCATCCTGGATGTCGTCTC	CCAGCTCATAGTACAGCATTGACC
Acadm	GCAGGGTCCTGAGAAGTGTTC	GCTTGTGAGCCGCTTTCG
Acta2	GTTCAGTGGTGCCTCTGTCA	ACTGGGACGACATGGAAAAG
Arg1	AACACTCCCCTGACAACCAG	CCAGCAGGTAGCTGAAGGTC
Atgl	GCAACCTGTCATTGTTCTAGC	CCAACGCCACTCACATCTAC
Atp5b	CACCAAGAAGGGATCGATCAC	GCAGGGTCAGTCAGGTCATCA
Ccl2	CCTTTTCCACAACCACCTCAAG	TAATTAAGGCATCACAGTCCGAGTC
Cd206	ATCCACTCTATCCACCTTCA	TGCTTGTTCATATCTGTCTTCA
Cd68	CTTCCCACAGGCAGCACAG	TGTAGCCTTAGAGAGAGCAGGTCA
Cldn5	GTGGAACGCTCAGATTTCAT	TGGACATTAAGGCAGCATCT
Col1a1	TAGGCCATTGTGTATGCAGC	ACATGTTCAGCTTTGTGGACC
Cpt1a	GAAGAAGAAGTTCATCCGATTCAAG	GATATCACACCCACCACCACG
Cpt1b	AGGGCTGCACTCCTGGAAG	CACGATAAGCCAGCTGGAGG
Cpt2	ACCATGCACTACCAGGACAGC	TGTCTTCAAGTTTAGGGATAGGCA
CytC	GGTGATGTTGAAAAAGGCAAGAA	TTGCCTCCCTTTTCCACAGT
Fabp1	CCCTTGATGTCCTTCCCTTTC	TTGCCACCATGAACTTCTCC
Fat/Cd36	GATGACGTGGCAAAGAACAG	TCCTCGGGGTCCTGAGTTAT
Fatp4	TGAAATCACCGCAGACGACAGG	GCTTGTCACCATCTCGTTTTCTC
Fasn	AGTCAGCTATGAAGCAATTGTGGA	CACCCAGACGCCAGTGTTC
Hmgcr	CTTGTGGAATGCCTTGTGATTG	AGCCGAAGCAGCACATGAT
Hsl	GCTGGGCTGTCAAGCACTGT	GTAACTGGGTAGGCTGCCAT
Il1b	CCTCAATGGACAGAATATCAACCAA	TCTCCTTGTACAAAGCTCATGGAG
Ldlr	AGGCTGTGGGCTCCATAGG	TGCGGTCCAGGGTCATCT
Lpl	AGCAGACGCGGGAAGAGA	AAGGTCTTGCTGCTGTGGTTG
Lxrα	AGGAGTGTCGACTTCGCAAA	CTCTTCTTGCCGCTTCAGTTT
Lxrβ	AAGCAGGTGCCAGGGTTCT	TGCATTCTGTCTCGTGGTTGT
Mmp9	CTCGAGGGCTTCCCTCTGA	GGCTGGAGGCCTTGGGT
Mmp13	AGGCCTTCAGAAAAGCCTTC	TCCTTGGAGTGATCCAGACC
Mttp	TCAGGAAGCTGTGTCAGAATGAAG	TTTCAAGTCCTCCCAGGATCA
Npc1l1	AGATCCCAACTTTGAGGTCTTCC	ACCGTCAGGTATTGCTGGTAGAAC
Ocln	TTGAAGAGTGGGTTAAAAATGTGTCT	TCAACTCTTTCCGCATAGTCAGAT
Pdk4	CCAGGGAGGTCGAGCTGTT	TGTCAATCTCCTTCAGGATATTGG
Pgc1α	TTGATGCACTGACAGATGGAG	GCTGAGTGTTGGCTGGT
Scarb1	TCCCCATGAACTGTTCTGTGAA	TGCCCGATGCCCTTGA
Scd1	CAGTGCCGCGCATCTCTAT	CAGCGGTACTCACTGGCAGA
Srebp1c	GGAGCCATGGATTGCACATT	GGCCCGGGAAGTCACTGT
Tfam	GGTCGCATCCCCTCGTCTA	GGATAGCTACCCATGCTGGAAA
Tgfb	GCAGTGGCTGAACCAAGGA	AGAGCAGTGAGCGCTGAATC
Tnfa	CTGAGGTCAATCTGCCCAAGTAC	CTTCACACAGCAATGACTCCAAAG
Zo-1	AGGACACCAAAGCATGTGAG	GGCATTCCTGCTGGTTACA

Supplementary Table 2
List of antibodies used in the study.

Antibody	Company	Catalogue	Host	Dilution
Pgc1α	Abcam	LS-C415095	Rabbit	1:1000
F4/80	Cell Signaling	D2S9R	Rabbit	1:200

Supplementary References

[1] Moschetta A, Frederik PM, Portincasa P, vanBerge-Henegouwen GP, van Erpecum KJ. The incorporation of cholesterol in sphingomyelin- phosphatidylcholine vesicles has profound effects on detergent-induced phase transitions. J Lipid Res 2002;43:1046-1053.

[2] Lopez CA, de Vries AH, Marrink SJ. Molecular mechanism of cyclodextrin mediated cholesterol extraction. PLoS Comput Biol 2011;7:e1002020.

[3] Bellafante E, Morgano A, Salvatore L, Murzilli S, Di Tullio G, D'Orazio A, et al. PGC-1beta promotes enterocyte lifespan and tumorigenesis in the intestine. Proc Natl Acad Sci U S A 2014;111:E4523-4531.

[4] Contursi A, Arconzo M, Cariello M, Piglionica M, D'Amore S, Vacca M, et al. let-7e downregulation characterizes early phase colonic adenoma in APCMin/+ mice and human FAP subjects. PLoS One 2021;16:e0249238.

[5] Huber W, Carey VJ, Gentleman R, Anders S, Carlson M, Carvalho BS, et al. Orchestrating high-throughput genomic analysis with Bioconductor. Nat Methods 2015;12:115-121.

[6] Hicks SC, Okrah K, Paulson JN, Quackenbush J, Irizarry RA, Bravo HC. Smooth quantile normalization. Biostatistics 2018;19:185-198.

[7] Ritchie ME, Phipson B, Wu D, Hu Y, Law CW, Shi W, et al. limma powers differential expression analyses for RNA-sequencing and microarray studies. Nucleic Acids Res 2015;43:e47.

[8] Zhou Y, Zhou B, Pache L, Chang M, Khodabakhshi AH, Tanaseichuk O, et al. Metascape provides a biologist-oriented resource for the analysis of systems-level datasets. Nat Commun 2019;10:1523.

[9] Kleiner DE, Brunt EM, Van Natta M, Behling C, Contos MJ, Cummings OW, et al. Design and validation of a histological scoring system for nonalcoholic fatty liver disease. Hepatology 2005;41:1313-1321.

JOURNAL
OF HEPATOLOGY

Journal of Hepatology

CTAT methods

Tables for a "Complete, Transparent, Accurate and Timely account" (CTAT) are now mandatory for all revised submissions. The aim is to enhance the reproducibility of methods.

- Only include the parts relevant to your study
- Refer to the CTAT in the main text as 'Supplementary CTAT Table'
- Do not add subheadings
- Add as many rows as needed to include all information
- Only include one item per row

1.1 Antibodies

Name	Citation	Supplier	Cat no.	Clone no.
Pgc1α		Abcam	ab191838	Polyclonal
F4/80		Cell Signaling	#70076	D2S9R

1.2 Organisms

Name	Citation	Supplier	Strain	Sex	Age	Overall n number
Wild type	-	Jackson Laboratory	C57BL6/J	Male	8 weeks	40
Pgc1α$^{fl/fl}$	PMID: 22087241	Anastasia Krallli	C57BL6/J	Male	8 weeks	100
Vil1-Cre	-	Jackson Laboratory	C57BL6/J	Female	8 weeks	2
iPgc1α$^{-/-}$	-	-	C57BL6/J	Male	8 weeks	100

1.3 Deposited data

Name of repository	Identifier	Link
Gene Expression Omnibus	GSE227610	www.ncbi.nlm.nih.gov/geo
Gene Expression Omnibus	GSE113819	www.ncbi.nlm.nih.gov/geo

1.4 Software

Software name	Manufacturer	Version
GraphPad Prism	GraphPad Software	v9.0
QuantStudio Design & Analysis	Applied Biosystems	V1.3.1
RStudio	RStudio, PBC	2022.07.2+576
Aperio Image Scope	Leica Biosystems	12.4.3.5008

1.5 Please provide the details of the corresponding methods author for the manuscript:

antonio.moschetta@uniba.it
